# Psychopathic and autistic traits differentially influence the neural mechanisms of social cognition from communication signals

**DOI:** 10.1038/s41398-022-02260-x

**Published:** 2022-11-29

**Authors:** Christine L. Skjegstad, Caitlyn Trevor, Huw Swanborough, Claudia Roswandowitz, Andreas Mokros, Elmar Habermeyer, Sascha Frühholz

**Affiliations:** 1 Department of Psychology, University of Oslo, Oslo, Norway; 2 Cognitive and Affective Neuroscience Unit, University of Zürich, Zürich, Switzerland; 3 Faculty of Psychology, Fern Universität Hagen, Hagen, Germany; 4 Clinic for Forensic Psychiatry, Psychiatric University Hospital Zurich, Zurich, Switzerland; 5 Neuroscience Center Zurich, University of Zurich and ETH Zurich, Zurich, Switzerland

**Keywords:** Neuroscience, Psychology

## Abstract

Psychopathy is associated with severe deviations in social behavior and cognition. While previous research described such cognitive and neural alterations in the processing of rather specific social information from human expressions, some open questions remain concerning central and differential neurocognitive deficits underlying psychopathic behavior. Here we investigated three rather unexplored factors to explain these deficits, first, by assessing psychopathy subtypes in social cognition, second, by investigating the discrimination of social communication sounds (speech, non-speech) from other non-social sounds, and third, by determining the neural overlap in social cognition impairments with autistic traits, given potential common deficits in the processing of communicative voice signals. The study was exploratory with a focus on how psychopathic and autistic traits differentially influence the function of social cognitive and affective brain networks in response to social voice stimuli. We used a parametric data analysis approach from a sample of 113 participants (47 male, 66 female) with ages ranging between 18 and 40 years (mean 25.59, SD 4.79). Our data revealed four important findings. First, we found a phenotypical overlap between secondary but not primary psychopathy with autistic traits. Second, primary psychopathy showed various neural deficits in neural voice processing nodes (speech, non-speech voices) and in brain systems for social cognition (mirroring, mentalizing, empathy, emotional contagion). Primary psychopathy also showed deficits in the basal ganglia (BG) system that seems specific to the social decoding of communicative voice signals. Third, neural deviations in secondary psychopathy were restricted to social mirroring and mentalizing impairments, but with additional and so far undescribed deficits at the level of auditory sensory processing, potentially concerning deficits in ventral auditory stream mechanisms (auditory object identification). Fourth, high autistic traits also revealed neural deviations in sensory cortices, but rather in the dorsal auditory processing streams (communicative context encoding). Taken together, social cognition of voice signals shows considerable deviations in psychopathy, with differential and newly described deficits in the BG system in primary psychopathy and at the neural level of sensory processing in secondary psychopathy. These deficits seem especially triggered during the social cognition from vocal communication signals.

## Introduction

Psychiatric disorders and psychiatric-affine personality dimensions can severely affect the recognition of social signals [1–4]. This is most evident for psychiatric disorders and dimensions with core social cognition deficits, such as in individuals with high-level psychopathic traits [5, 6]. These deficits in psychopathy are commonly accompanied by deviant functional brain activations in the social brain network as potential neural markers for such psychiatric conditions [7–9]. The social brain network includes several cortical and subcortical subsystems [10–12]. The mirror neuron subsystem (MNS) allows action observations in others (IFC inferior frontal cortex, IPS intraparietal sulcus), the mentalizing subsystem enables reflection of others and the self (STC superior temporal cortex, TPJ temporo-parietal junction, dMFC dorsal medial frontal cortex), the empathy subsystem triggers emotional and cognitive contagion (aINS anterior insula, ACC anterior cingulate cortex), and a limbic subsystem decodes social salience and socio-affective values (Amy amygdala, MTL medial temporal lobe, vMFC ventral medial frontal cortex, OFC orbitofrontal cortex).

Individuals with psychopathic traits show dysfunctional brain activity mainly in cognitive social brain nodes and their connections [13, 14]. Data about these neural dysfunctions however have been somehow limited and partly inconsistent, probably based on several factors [7, 15]. A first factor concerns the subtypes of psychopathy that show phenotypical and neurofunctional diversity [16, 17]. The primary and probably most prototypical subtype or psychopathy factor (“instrumental social exploitation” subtype, idiopathic psychopathy) involves low-anxious traits, manipulative and callous behavior, and increased self-focus. While socially unresponsive to others’ distress (amygdala and aINS hypoactivity) [7, 16, 18–20], social affection (MTL dysfunctions) [19], and coercive harm (dMFC hypoactivity) [21], their cognitive empathy abilities (MNS system, neural mentalizing system) [22] seem instrumental to manipulate others [23, 24] and to enjoy others’ pain (ventral striatum (vStr) hyperactivity) [19, 25] and violence (OFC, aINS, dMFC hyperactivity) [26]. The secondary subtype (“antisocial deviance” subtype) involves high anxiety [23], impulsivity (dlPFC dysfunctions) [27–29], and primary reward dependency (vStr hyperactivity) [30]. This subtype shows emotional reactivity (amygdala) and empathic concern (aINS) to others’ distress [7, 19, 31] that seems however not to be of a socially rewarding nature (SN substantia nigra, vStr, vmPFC hypoactivity) [16]. Overall, this indicates differential patterns of abilities and deficits in social cognition as well as of neural decoding of social signals across psychopathy subtypes, which might also explain contradictory findings about limbic system activity [15].

A second factor concerns the focus on visual material that carries specific feature information as the target focus of social recognition tasks for participants. First, visual material and especially face recognition setups are often limited to receptive social cognition tasks that only have a restricted interactive and communicative component. Unlike human faces, the human voice seems more strongly related to communication contexts, as it is the carrier for nonverbal (non-speech) and speech communications [32, 33]. Voice communication has specific requirements for social cognition, as it is more embedded in complex social interactions [32] and has temporal structure properties [34]. This might be relevant for the distinction of psychopathy subtypes and related communication styles [35], and might point to the relatively unexplored relevance of the dorsal striatum (basal ganglia [BG] system) as a social and communicative pattern decoding node [34, 36, 37]. Second, the more fundamental abilities in psychopathic individuals to discriminate social from non-social signals have been largely unexplored. This is however the most fundamental social cognition ability and is generally relevant to distinguish communicative voice and speech signals from other non-human sounds [38]. This social from non-social sound separation is accomplished by sensory brain systems in the auditory cortex (AC) [38]. In neurotypicals, neural “voice areas” (VA) in the STC have been identified that support voice and speech sound detection [39, 40], and given their social voice signal detection properties, the VA neural patterns might be differentially impaired in psychopathy.

A third factor concerns the psychopathological and neurofunctional overlap with other psychiatric disorders and traits. Investigations tried to identify such commonalities and differences of psychopathy with cluster-B personality disorders [41, 42], adolescent conduct disorders [43], and autistic spectrum disorders (ASD) and traits [44, 45]. This was done as a means of understanding the mechanisms and differential origins of specific social deficits in psychopathy. Especially ASD and autistic traits seemed relevant here, as they share cognitive [44], neurofunctional [45], and neurobiological genetic links with psychopathy [5]. Individuals with high-level autistic traits have neurofunctional deficits in the social brain network [46], and share dysfunctions in the neural empathy [47–49] and limbic socio-affective networks [50, 51] with individuals scoring high on psychopathic traits. Opposite of psychopathy, however, people with ASD and autistic traits struggle with cognitive empathy but can feel affective empathy when they understand others’ emotions [52, 53].

Besides marked socio-affective deficits, about 90% of those with ASD are also affected by sensory processing symptoms [54]. The neural markers of ASD point to deficits already at the level of sensory processing, as social impairment severity correlates with auditory VA activation to voice and speech signals in ASD [55, 56], and the VA as a neural seed is under-connected to the frontal mentalizing and mirror network in VA [56]. An open research question thus seems if psychopathic traits are also marked by neural deficits in the sensory analysis of voice signals, given the overlap in social cognition deficits across both psychiatric phenotypes.

We here took a parametric neuroimaging approach to test several of the outlined open research questions about the neurofunctional mechanisms that underlie social perception abilities and deficits in psychopathy as a dimensional trait measure. We hypothesized that in response to social voice stimuli, psychopathic and autistic traits would differentially correlate with neurofunctional diversity in regions of the social cognitive and affective brain networks (i.e., mirror neuron, empathy, mentalizing, and limbic subsystems). The study was mostly exploratory, but with a specific focus on these networks.

## Materials and methods

### Participants

Participants were invited to take part in a functional magnetic resonance imaging experiment (fMRI) to quantify brain activity for auditory communication sound processing. The study included *N* = 113 human participants with an age range 18–40 y (mean age 25.59 y, SD 4.79) and including 41.6% male (*n* = 47) and 58.4% female participants (*n* = 66). We recruited as many participants as possible for the data set and did not include a stopping rule. We also did not perform an a priori for the sample size due to the exploratory nature of the design. The sample size allows for parametric regression analysis of a varied distribution and is at least equal or higher in comparison to the sample sizes of similar studies [57, 58]. All participants had normal hearing and normal or corrected-to-normal vision. Exclusion criteria were having hearing impairments, psychiatric or neurological disorders in life history. Only two participants were left-handed. Each participant gave informed and written consent for their participation following the ethical and data security guidelines of the University of Zürich (Switzerland). The experiment was approved by the cantonal ethics committee of the Swiss canton of Zürich.

### Communication sound processing experiment

**Fig. 1:**
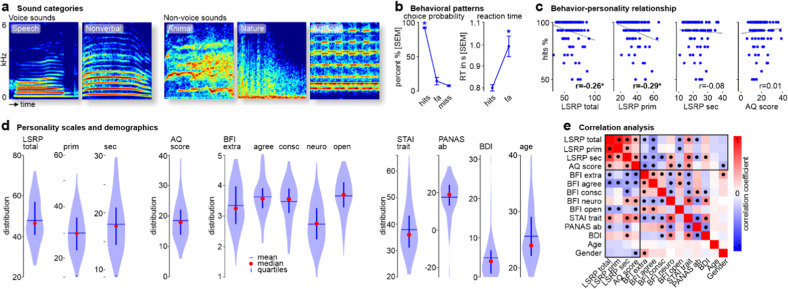
Experimental sounds, behavioral performance, and personality traits. **a** Spectrograms of example sounds from the five sound categories, namely voice (speech, nonverbal) and non-voice sounds (animal, nature, artificial). **b** Choice probability and reaction times for the sound repetition task separately for hit trials (correct detection of repetitions), false alarms (fa, incorrect classification of non-repetition trials as a repetition), and missed trials (miss, missed detection of repetition trials); *p < 0.05 (Bonferroni corrected). **c** Correlation of detection probability for hit trials and psychopathic (LSRP total score, LSRP primary score [prim], LSRP secondary score [sec]) and autistic traits (AQ score); *p < 0.05 (FDR corrected). **d** Distribution of scores for personality traits (LSRP, AQ, BFI, STAI, PANAS, BDI) and demographic features (age). **e** Correlation matrix (mirror symmetric) between personality traits and demographic scores (age, gender); ● *p* < 0.005 (FDR corrected). AQ Autism Spectrum Quotient, BDI Beck Depression Inventory, BFI Big Five Inventory (extra extraversion, agree agreeableness, consc conscientiousness, neuro neuroticism, open openness), LSRP Levenson Self-Report Psychopathy scale, PANAS Positive and Negative Affect Schedule (ab affective balance), STAI State Trait Anxiety Inventory.

For the communication sound processing experiment, we used a set of 500 ms natural sound recordings consisting of 70 human voice sounds (speech, non-speech vocalizations) and 70 non-voice sounds (animal, artificial, natural sounds). All sound files had a duration of 500 ms. All sounds were normalized to the root mean square (RMS) and were presented at 70 dB sound pressure level (SPL) during the experiment. See Fig. 1a for more details on stimuli characteristics.

The five voice and non-voice sound conditions were presented in a pseudo-random order (no more than three repetitions of sounds from the same category) with an inter-stimulus interval between 3 and 5 s. Each of the 140 sounds was played once with randomly chosen repetitions in 10% of trials with a total of 14 repetitions. Participants were asked to perform a one-back task and were instructed to press a button on a button box with their right index finger to indicate when a sound was repeated from the previous trial.

### Behavioral data analysis

The behavioral performance of the sound repetition detection task was quantified with reaction times and detection accuracy measures. Reaction time and detection accuracy were quantified for hit trials (repetition present, repetition detected) and false alarm trials (no repetition, repetition detected), with the percentage of miss trials (repetition present, repetition not detected) also quantified. These measures were subjected to a one-way repeated-measures ANOVA to estimate significant differences between conditions. The significance threshold was set to *p* = 0.05.

We also performed Pearson correlation analyses between individual reaction time and detection accuracy scores and the psychopathic and autistics trait scores. Significant correlations were determined at *p* = 0.05 (FDR corrected to account for multiple testing).

### Preprocessing and statistical analysis of brain data

Structural and functional brain data were acquired on a 3 T Philips Ingenia MR scanner, and brain data preprocessing and analysis followed standard procedures. Preprocessing and statistical analyses of functional images were performed using the Statistical Parametric Mapping software (SPM12, Department of Cognitive Neurology, London; http://www.fil.ion.ucl.ac.uk/spm). Functional data were first manually realigned to the AC-PC axis and then motion corrected with realignment to the mean functional image, followed by slice time correction. Each participant’s anatomical T1 image was then co-registered to the mean functional EPI image and segmented for estimating the normalization parameters. The anatomical and functional images were then normalized to the Montreal Neurological Institute (MNI) stereotactic space (http://www.mrccbu.cam.ac.uk/Imaging/mnispace.html) while functional images were re-sampled into an isotropic 2 mm^3^ voxel size. All functional images were spatially smoothed at 8 mm full width half maximum (FWHM) isotropic Gaussian kernel.

Functional brain data were entered into a fixed-effects single-subject analysis, with a general linear model (GLM) design matrix containing five separate regressors for each of the conditions plus an additional regressor for all repetition trials, that further included six motion correction parameters as regressors of no interest to account for head motion artifacts. All trials were modeled with a stick function aligned to the onset of each stimulus, which was then convolved with a standard hemodynamic response function (HRF).

Contrast images for the five sound conditions were then taken to several separate random- effects factorial group-level analyses. First, we performed contrasts between conditions to determine functional brain activity patterns that were associated with general ([all voice > non-voice sounds]) and specific voice sound processing ([speech > non-voice], [non-speech > non-voice], [speech > non-speech], and [non-speech > speech]). Second, we performed a regions-of-interests (ROI) analysis of significant clusters resulting from the first factorial analysis. Third, we finally performed separate whole-brain analyses to quantify the individual association between neural effects for voice processing with trait psychopathic and autistic scores across all brain voxels (see Supplemental methods for more information).

### Psychopathic and autistic trait assessment

After scanning, the participants sat at a computer where they completed various questionnaires. For assessing psychopathic and autistic traits, we included established and validated scales for the self-report assessment of participants that could be used in an online format [59]. To self-assess psychopathic traits, we used the Levenson Self-Report Psychopathy Scale (LSRP; LSRP_total_ as total score) [28], which seems a reasonable scale for self-reports on individual psychopathy in non-institutionalized populations [60, 61], with good construct and concurrent validity [62], and the subscales have convergent validity as higher scores are prototypical for psychopathy ratings [60]. The LSRP shows associations with the neurobiological [5] and neuroimaging markers of psychopathy [63] in terms of structural [64] and functional brain measures [14, 26]. The LSRP is a 26-item questionnaire that predominantly assesses primary (LSRP_prim_, 16 items) and secondary psychopathy (LSRP_sec_, 10 items) [62]). LSRP data were acquired here with a parametric 5-point Likert scale, but arithmetically converted to 4-point Likert scale dimension for comparability with previous studies [26, 62]. The points were converted using the following formula:

$${\mathrm{New}}\;4pt\;{\mathrm{Score}} = \frac{{\left( {B - A} \right) \times ({\mathrm{Original}}\;5pt\;{\mathrm{Score}} - a)}}{{b - a}} + A$$

Where a = original scale minimum (for a 5-point Likert, 1), b = original scale maximum (for a 5-point Likert, 5), A = new scale minimum (for a 4-point Likert, 1), and B = new scale maximum (for a 4-point Likert, 4). Preliminary reports might suggest a three-factor structure of the LSRP [65], but without consistent evidence yet. The three factors do not show improved reliability or validity, share associations with one another, are rather theoretical, and need improvements [65–68]. Because the two-factor model is adequate, established, and shows convergent and discriminant validity with other variables, it seems still be the best use of the LSRP [68].

The other major personality dimension of interest in our study were autistic traits. A commonly used scale to measure autistic traits in a self-report format is the Autism-Spectrum Quotient (AQ) test [69]. The AQ is a 50-item questionnaire that quantitively measures the extent and degree to which an adult with normal intelligence may have traits associated with the autism spectrum [69]. The AQ assesses five different domains such as social skills, attention switching, attention to detail, communication, and imagination. Traits associated with autistic-like behavior are poor social and communication skills, poor imagination, exceptional attention to detail, and poor attention-switching/ strong focus of attention. The AQ is proven to have adequate construct validity, convergent validity with related measures and can differentiate people with and without autistic traits [70–72]. The AQ score is also associated with differences in structural [73, 74] and functional brain patterns [75, 76].

We also quantified additional common personality traits (extraversion, agreeableness, conscientiousness, neuroticism, and openness) using the Big Five Inventory (BFI), negative and positive affect (Positive and Negative Affect Schedule, PANAS), as well as affective balance (PANAS_ab_; difference score between negative and positive affect) trait dimensions of anxiety (State-Trait-Anxiety Inventory, STAI_trait_), and depression levels (Beck Depression Inventory, BDI-IA) (see Supplemental Methods).

## Results

### Variability of psychopathic and autistic traits in a human community sample

We found a broad variability and individual differences in the assessed psychopathic, autistic, and general personality traits (Fig. 1d), with Cronbach’s alpha being in an acceptable range of scale reliability (LSRP_total_ 0.84, LSRP_prim_ 0.84, LSRP_sec_ 0.70, AQ score 0.78, BFI_extra_ 0.81, BFI_agree_ 0.74, BFI_consc_ 0.77, BFI_neuro_ 0.78, BFI_open_ 0.77. PANAS_ab_ 0.88, STAI_trait_ 0.90, BDI 0.88). Given the two-factor model of psychopathy, the LSRP_total_ score accordingly positively correlated both with LSRP_prim_ (Pearson correlations, *n* = 113; r = 0.909, *p* < 0.001; FDR corrected) and LRSP_sec_ (r = 0.726, *p* < 0.001), and both psychopathy factors also showed some interdependency (r = 0.373, *p* < 0.001) (Fig. 1e, Table. S1). From the three psychopathy scores, only LSRP_sec_ showed a positive correlation with the AQ score (r = 0.356, *p* < 0.001).

Besides these interdependencies within psychopathic and autistic traits, both personality dimensions also showed common and differential positive and negative associations with broader socio-affective personality traits (Fig. 1e). First, all four scores for the psychopathic and autistic traits (LSRP_total_, LSRP_prim_, LSRP_sec_, AQ score) correlated negatively with agreeableness (r’s < −0.349, *p*’s < 0.001) as quantified by the Big Five Inventory (BFI). Second, we found a negative correlation pattern overlap between LSRP_prim_ and the AQ score only on the BFI factor of openness for experiences (r < −0.231, *p* < 0.001). Third, we found more extensive correlation pattern overlaps between LSRP_sec_ and the AQ score, with negative correlation overlaps on the BFI factor extraversion (r < −0.243, *p* < 0.001), and a positive correlation overlaps on the BFI factor neuroticism (r > 0.153, *p* < 0.001) and STAI trait anxiety (r > 0.384, *p* < 0.001). Fourth, specific correlations were found for LSRP_sec_, inducing a negative correlation with PANAS_ab_ (r = −0.288, *p* < 0.001) and a positive correlation with the BDI score (r = 0.266, *p* < 0.001) reflecting high negative emotionality. Furthermore, the factor gender correlated negatively with autistic traits (r = −0.278, *p* < 0.001) indicating higher autistic trait scores in male participants. No significant correlations were found with the factor age.

### Performance in the sound repetition detection task is associated with psychopathic traits

All participants were very accurate in indicating the actual repetition of sounds (hit trials, mean 92.21%), and this was significantly (F_1,2_ = 196.170, *p* < 0.001, Greenhouse Geiser [GG] corrected) above the percentage of false alarms (no sound repetition, but button press; mean 14.41%) and miss trials (sound repetition, but no button press; mean 7.79%). In terms of reaction times, we found that individuals took more time to respond in false alarms trials than in hit trials (F_1,1_ = 6.349, *p* = 0.016).

We furthermore determined whether the accuracy of hit rates for repeated sounds would depend on the four major personality traits investigated here. We accordingly found a negative correlation (linear partial correlation controlling for age and gender, *n* = 113, FDR corrected) between the LSRP_total_ (r = 0.257, *p* = 0.014) and the LSRP_prim_ score (r = −0.292, *p* = 0.009) with the individual hit rate level. This indicates that individuals with high levels of psychopathic traits and especially individuals scoring high on primary psychopathy have more difficulties in detecting sound repetitions.

### Brain activity in the neural voice processing network differentially depends on psychopathic and autistic traits

**Fig. 2:**
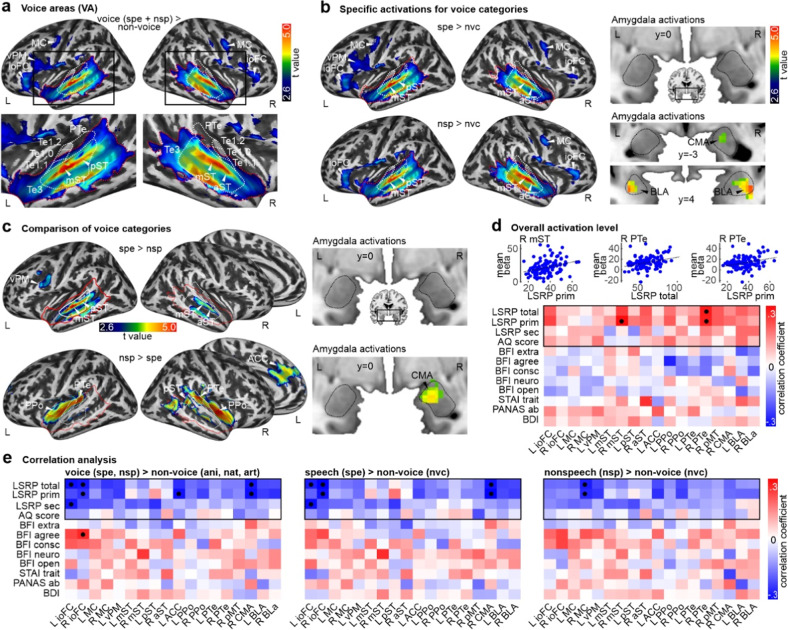
Functional activations for voice compared to non-voice sounds. **a** Bilateral frontal (IFC, MC) and temporal cortex activity (mST, pST) for voice compared to non-voice sound (*n* = 113). Lower panel shows an enlarged view of the section with the black rectangle in the upper panel, indicating peak locations in the AC (auditory cortex) located in the higher-order auditory area Te3. Dashed white outlines indicate primary AC (Te10–1.2), secondary AC (PTe), and higher-order AC (Te3); red dashed outline indicates the temporal voice area (VA). All activations are thresholded at a combined voxel threshold *p* < 0.005 and cluster level threshold k > 55, resulting at *p* < 0.05 corrected at the cluster level. **b** Functional activity (*n* = 113) separately for the voice sound categories, namely speech sounds (spe) and non-speech sounds compared against non-voice sounds (nvc). Left panels show cortical activity, right panels show activity in the amygdala and its subregions (*CMA* centromedial amygdala, *BLA* basolateral amygdala). **c** Functional activity (*n* = 113) for comparing voice sound categories against each other, namely speech (spe) against non-speech sound (nsp) (upper panel), or vice versa (lower panel). **d** Correlations between personality traits and mean brain activity to all 5 sound categories (*n* = 113). The upper panel shows the three significant correlations between activity in peak activity locations (as derived from **a**–**c**) and LSRP total and primary score; lower panel shows all correlations (● *p* < 0.05, FDR corrected). **e** Pearson correlation (*n* = 113) between personality traits and difference scores of peak activity locations for comparing voice against non-voice sounds (left panel), speech against non-voice sounds (mid panel), and non-speech against non-voice sounds (right panel). ACC anterior cingulate cortex, aST anterior superior temporal cortex, BLA basolateral amygdala complex, CMA centromedian amygdala complex, ioFC infero-orbital frontal cortex, MC motor cortex, mST mid superior temporal cortex, pMT posterior middle temporal cortex, PPo planum polare, PTe planum temporale, pST posterior superior temporal cortex, vPM ventral premotor cortex, nsp non-speech sounds, nvc non-voice sounds, spe speech sounds, voc voice sounds.

We presented voice and non-voice sounds in the sound processing experiment and first determined neural activation patterns for voice sounds in comparison with non-voice sounds (Fig. 2a, Table. S8). Across the whole sample of participants, voice sounds elicited higher and spatially extended activity in bilateral AC, with peak activations mainly found in the higher-order AC Te3 region. Additional activity was found on frontal cortex, with peaks located in motor cortex (MC), ventral premotor areas (vPM), and infero-orbital frontal cortex (ioFC). This activity pattern resembles commonly found neural voice processing nodes [40] and MC peaks were in areas innervating the lips [77].

This neural activation pattern for processing voice signals was largely repeated when we determined the neural effects specifically for processing speech and non-speech voice signals (Fig. 2b, Table. S9). The neural effects revealed an almost identical pattern as for voice processing in general, while the neural effects for non-speech sounds somehow differed from this overall pattern. Non-speech sounds did not activate the left motor and premotor areas, but they showed some specific activations in the ACC as well as left and right amygdala, presumably located in the centromedian (CMA) and basolateral subnuclei (BLA) of the amygdala complex. We additionally compared neural effects for speech and non-speech sounds directly against each other (Fig. 2c, Table. S10) and found that speech signals specifically activate higher-order AC and left vPM, while non-speech sounds specifically activate secondary AC regions (planum temporal *PTe*, planum polare *PPo*) and right amygdala (CMA subnucleus).

In the next step, we determined whether personality traits showed an association with the neural activation levels across all five sound categories as a general measure of sound sensitivity (Fig. 2d, Table. S2). For this purpose, we quantified the mean activation in regions of interest (ROIs) that showed significant activation in the above-reported analysis (Fig. S1). We found that LRSP_total_ (Pearson correlations, *n* = 113, r = 0.269, *p* = 0.028, FDR corrected) and LSRP_prim_ (r = 0.264, *p* = 0.028) were positively correlated with activity in right PTe and LSRP_prim_ showed an additional positive association (r = 0.287, *p* = 0.025) with an area in the mid-section of right STC (mST) as part of higher-order AC. No associations were found between the overall activation level in these ROIs and the AQ score. We also assessed whether personality traits showed an association with activation levels for voice processing as a social sound signal (Fig. 2e, Figs. S3–5). When quantifying the activity difference for voice against non-voice sounds in the ROIs, we found several associations with the LSRP scores (Pearson correlations, *n* = 113, FDR corrected). Left and right ioFC showed lower signals with higher LSRP_total_ scores (r’s < −0.272, *p* < 0.040), where left ioFC activity was negatively associated with LSRP_sec_ (r = −0.276, *p* = 0.019) and right ioFC was negatively associated with LSRP_prim_ (r = −0.240, *p* = 0.041). Furthermore, right CMA activity was negatively associated with LSRP_total_ (r = −0.275, *p* = 0.019) and LSRP_prim_ (r = −0.286, *p* = 0.019), with LSRP_prim_ also showing a negative association with ACC activity (r = −0.279, *p* = 0.033). This pattern of brain-personality associations was largely repeated for the neural effects of speech processing (r’s < −0.268, *p*’s < 0.024), with only the ACC-LSRP_prim_ association missing. For the neural effects of non-speech sounds, we only found negative relationships with the right MC (r’s < −0.257, *p* < 0.035).

### Psychopathic and autistic traits show largely differential associations with neural voice processing

**Fig. 3:**
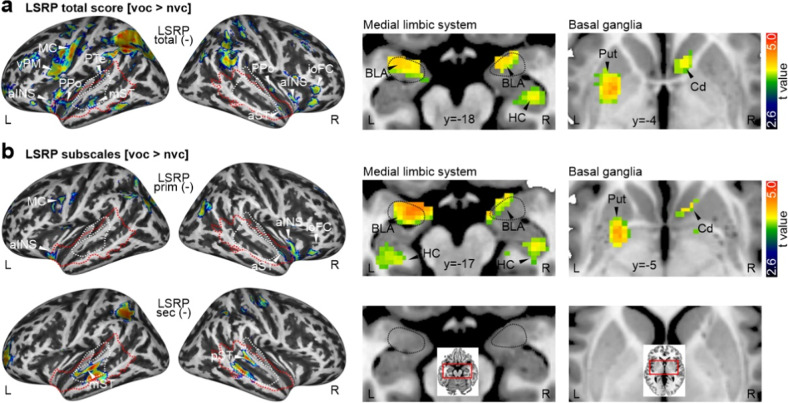
Neural voice processing depending on psychopathic traits. **a** Increased cortical activity (left panels) as well as subcortical activity in the medial limbic system (BLA basolateral amygdala, HC hippocampus) and the basal ganglia (Put putamen, Cd caudate nucleus) for voice compared to non-voice sound with decreasing levels of the LSRP total score (*n* = 113). **b** Same analysis as in a, but separately for LSRP primary (upper panel) and secondary factor (lower panel) (*n* = 113).

We performed several multiple regression analyses to assess if there are further influences of psychopathic and autistic traits on neural voice processing in a broader brain network, firstly with the different LSRP scores (Fig. 3). In these models, we also included the additional personality scores (BFI, STAI_trait_, PANAS_ab_, BDI) and the demographic variables (age, gender) as regressor-of-no-interest to account for the potential influence of these variables. Using the LSRP_total_ score as a predictor for neural signal difference between voice and non-voice sounds (Fig. 3a, Table. S11), we observed several negative associations with a widespread brain network consisting of low-order AC (PPo, PTe) and higher-order AC (mST, anterior STC aST), motor (motor cortex MC) and premotor regions (ventral premotor cortex vPM), a fronto-insular system (ioFC, aINS), medial limbic system (BLA, hippocampus HC), and BG system (putamen Put, caudate nucleus Cd). Some of these negative associations were also repeated when using the LSRP_prim_ score as a predictor (Fig. 3b, Table. S12). The same analysis was also performed with comparing the specific voice subcategories (speech, non-speech) against non-voice sounds (Fig. S4), with some differential laterality effects for speech and non-speech sounds in association with LSRP scores.

**Fig. 4:**
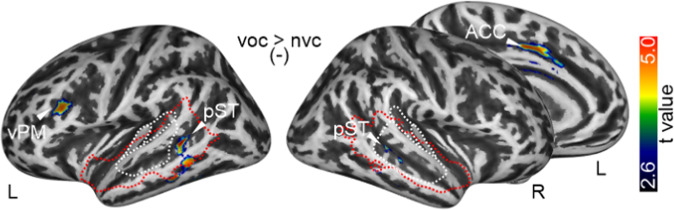
Neural voice processing depending on autistic traits. Increased cortical activity (left panels) for voice compared to non-voice sounds with decreasing levels of the AQ score (*n* = 113).

We then performed the same multiple regression analysis with the AQ score as the predictor variable and the other personality scores as regressors of no interest (Fig. 4, Table. S13). We found that only a small number of regions were negatively associated with autistic personality scores and their influence on neural voice processing. We found negative association specifically in bilateral higher-order AC (posterior STC pST), left vPM, and ACC. The same analysis was also performed for the neural processing of speech and non-speech sounds, with speech sound showing a specific association with a larger region in pST (Fig. S5). It seemed like a part of this negative activation pattern was overlapping with the pattern found for the LSRP score.

To directly investigate some potential activation overlap here between predictions by LSRP and AQ score, we computed a final model including both LSRP and AQ score in order to perform a conjunction analysis using the conjunction null hypothesis [78] to identify significantly similar patterns. None of the conjunction analyses revealed any significant neural activations, thus pointing to rather differential influences of psychopathic and autistic traits on the neural voice processing.

## Discussion

As a first important finding, our sample included individuals with a broad distribution of low to high psychopathic and autistic scores, indicating that our sample covered a broad range of these trait phenotypes. This broad distribution accordingly also involved participants with trait scores close to or within a potential range of clinical significance. Out of the total 113 participants, 14 (12.4%) participants met the screening cutoff point of ≥26 of the AQ score, which may indicate the possibility of psychopathological problems in the range of an ASD diagnosis, and 4 (3.5%) participants even met the clinical threshold of ≥32 indicating severe autistic complications [69]. The LSRP scores seemed to resemble the scores originally reported by Levenson and colleagues for a community sample [28]. While there seems no designated cutoff score for the LSRP [79], Brinkley and colleagues [62] have previously used a cutoff score of 58 from the LSRP total score to identify psychopathic participants from a sample of imprisoned offenders in a US state prison. This cutoff score was mainly used for data analytical purposes and considered participants in the top third of the distribution as “psychopathic”. One must be careful in applying such measurement and data analysis procedures across countries (USA and Switzerland) and study samples (imprisoned offenders, community sample), but when following this example of Brinkley and colleagues [62], 32 (28.3%) of our participants would meet the standard of being considered psychopathic in a clinically relevant range. While the numbers for autistic traits seem to be in accordance with previous estimations of these trait distributions in the general population [80], the cutoff score of 58 for the LSRP seems to overestimate the proportion of clinically relevant psychopathy in our community sample, which commonly seems to be in a range of 1.2–4.5% [81]. Given this common range, the cutoff in our sample would have been ~66–69 of the LSRP total score to achieve a similar proportion of participants with a clinically relevant psychopathy score.

After establishing the distribution of psychopathic and autistic trait scores in our sample, we performed correlation analyses to assess the relationship of these specific traits with more general personality traits (Fig. 1e). First, as expected, all psychopathy scores were positively correlated with each other, but only the secondary psychopathy subtype was positively associated with the autistic score, pointing to phenotypical commonalities between autistic personality and a psychopathy subtype [44, 47, 49]. Second, all psychopathic scores and the AQ score were negatively correlated with the BFI factor agreeableness [82, 83], which indicates a common disregard for and/or non-interest in social harmony. Although such disregard seems common with high psychopathic and autistic traits, the motivation for this might differ, either reflecting (anti-)social utilitarianism or social avoidance, respectively [49, 84, 85]. Third, overall psychopathy showed a profile of high trait anxiety, negatively shifted affective balance, low extraversion, and low openness. While negative affectivity and low extraversion follow a common description of psychopathy [5], the observation of low levels of openness for new experiences is rather surprising [82]. Psychopaths especially of the primary subtype have a tendency for sensation-seeking given some low arousability [86, 87], thus suggesting rather high levels of openness. However, openness in psychopathy is restricted to experiences that are instrumental to the individual, whereby unbiased experiences outside this instrumental effort might be avoided. Fourth, while primary psychopathy had only a low level of openness in common with the autistic trait, secondary psychopathy and the autistic trait had many more commonalities (high neuroticism and trait anxiety, low extraversion). This points to overall socio-affective impairments and antisocial tendencies in the phenotype of both dimensions and common social deficits that also have been suggested in previous reports [88]. Secondary psychopathy however showed additional associations with low conscientiousness, negative affective balance, and high depression scores [82], indicating that their psychopathological status can be more severe than in individuals with autistic traits.

Psychopathic and autistic traits thus seem to be associated with certain impairments along socio-affective personality traits. We next tested if this would also apply to brain mechanisms for the processing of socially relevant voice and speech communicative signals. First, we determined the general fronto-temporal-limbic neural network for processing such communicative signals independent of variations on the trait dimension. This network comprised low- and higher-order AC, (pre-)motor regions, and infero-orbital frontal regions as previously described [38–40], with additional amygdala and ACC activity during the processing of non-speech voice sounds given their higher socio-affective nature [89]. Within this broader neural network for voice and speech processing, we then tested if activity levels would depend on psychopathic and autistic trait scores. First, primary psychopathy was associated with overall higher neural activity in right secondary and higher-order AC during the processing of sounds in general (Fig. 2d). This points to increased reactivity to stimulation, which might be caused by a generally low arousal base rate in psychopathic individuals [87]. Second, when contrasting neural activity differences for voice against non-voice sound processing, no correlations were found with the autistic trait score in these ROIs from this general voice processing network (Fig. 2e). These relatively intact neural mechanisms for voice processing in individuals with high autistic traits is in line with recent observations [55], pointing to more subtle social voice processing deficits as we will discuss below. Third, unlike for the autistic trait, we found only negative correlations with increasing psychopathy scores when contrasting neural activity differences for voice against non-voice sounds processing, indicating relative neural hypoactivity during the processing of voice signals. These negative correlations revealed a specific pattern of results. Primary and secondary psychopathy showed a hypoactivity in the ioFC, especially during the processing of speech sounds, but with a laterality effect (Fig. 2e). The ioFC is part of the ventral auditory stream and supports speech recognition, with the left ioFC rather responding to the understanding of segmental speech and vocalization units [90], while the right ioFC might rather decode suprasegmental speech modulations [91] and their socio-affective associations [92]. Given that right ioFC activity correlated positively with the general personality trait of agreeableness, this points to considerable impairments for socio-affective processing from speech signals in primary psychopathy.

There were further observations of neural hypoactivity associated with primary psychopathy. Primary psychopathy revealed hypoactivity in right MC for the processing of non-speech voices and in right amygdala for speech sounds. Hypoactivity in the MC was in an area that innervates the lips [77] and might contribute to the motor mirroring of nonverbal vocalizations supporting the decoding of meaning from these signals [89], which could also be connected to the negative signal correlations with ACC activity in primary psychopathy. The amygdala is part of a socio-affective brain system for social cognition. Of note is that right amygdala activity for speech sound processing was not found in the general analysis independent of the trait analysis, suggesting that variations on the primary psychopathy score suppressed significant group activations. The amygdala peak was furthermore located in the centromedian subregion, which usually controls affective responding to social signals [93], and which again seemed to be neurally impaired in primary psychopathy.

While the former analysis was restricted to ROIs of the neural voice processing network, we performed additional and unrestricted whole-brain analyses to reveal the broader influence of psychopathic and autistic traits in the neural processing of social communication signals. We found that increasing levels of psychopathic traits were related to widespread hypoactivity across socio-affective processing networks (Fig. 3 and Fig. S4) that are typically implicated in social deficits in psychopathy. Specifically, we found hypoactivity in the MNS (IFC, IPS), mentalizing (TPJ, STC), empathy (aINS), and limbic networks for socio-affective evaluations and associations (Amy, HC, OFC). These large-scale dysfunctions in social brain systems indicate considerable social voice processing deficits, with voice signals being processed either in an unattached and antisocial manner [7, 94] or not being socially salient for such individuals [95]. Additional hypoactivity was found in the BG system, which has not yet been in the focus of the social brain theory [10–12]. The BG, however, seem important for decoding social and temporal information from auditory communication signals [34] and thus seem a central node for processing voice signals [37].

These results overall point to broad social processing deficits from voice communication signals in psychopathy, but there were also differential neural deficits for primary and secondary psychopathy. Primary psychopathy seems to show neural deficits in almost all social processing subnetworks and the additional BG subnetwork. This confirms the lack of affective empathy [96], a lack of emotional awareness of others in primary psychopathy [7], as well as specific blunted socio-affective responses toward the voice stimuli and a potential lack of motivation to listen [20, 97] or to engage with them [98]. Secondary psychopathy on the other hand seems to show intact empathic and affective (limbic) neural processing mechanisms but with deficits in the neural mirroring (IPS) and mentalizing subnetworks (STC). The latter hypoactivity in STC for secondary psychopathy might overlap with sensory and auditory object processing deficits in the voice-sensitive cortex accompanying or complementing the mentalizing problems. Sensory and auditory object processing deficits in psychopathy have not been reported before and seem to be a novel finding, as the psychopathological dynamic of psychopathy was previously assumed as a cognitive-emotional deficit [5, 99]. Such an upstream processing deficit at the level of sensory cortices (especially in higher-order AC Te3) could centrally contribute to the antisocial behavior patterns in secondary psychopathy. Sensory analysis and auditory object recognition of social signals might also be more relevant for auditory than for visual social signals, as auditory communication signals are more complex in nature and dynamically evolve over time. Surprisingly, we did not find a direct correlation between psychopathy scores and neural peaks of the voice processing network in the AC, pointing to some differential effects here that might also concern the neural effects that we found for autistic traits.

On the personality phenotype level, we described some similarities between secondary psychopathy and autistic traits above. Increasing autistic traits led to hypoactivity in left vPM, ACC, and in bilateral AC (Fig. 4). While the latter observation might indicate some neural overlap between deficits in secondary psychopathy and autistic traits, a statistical conjunction analysis revealed no significant common effects. This might indicate some form of common sensory deficits in secondary psychopathy and autistic traits, but at different levels of the sensory processing hierarchy. We must note again that neither secondary psychopathy nor autistic traits correlated with auditory cortical activity in the general voice processing network, and the activations in the whole-brain regression analyses seem to be somewhat off the auditory cortical peaks of the general voice processing network. It seemed that these activations were located one step downstream of the auditory processing hierarchy in the extended belt of the auditory cortical voice regions. These regions seem rather representing sensory feature integration than feature analysis [38, 55], and probably representing intermediate nodes of the ventral and dorsal auditory pathways to frontal regions [100]. The peak activations had stronger ventral stream focus (anterior-inferior ST) in secondary psychopathy pointing to auditory object classification deficits, and a stronger dorsal stream focus (pST) in autistic traits. The dorsal stream has a specific significance in processing voice signals in a communicative context [100]. Sensory integrative communitive deficits have been described for autistic individuals [101], especially for the neural processing of voices [56], but less in adults with high-functioning ASD [55]. In our community sample, we show that increasing autistic traits can indeed lead to abnormal neural functions during voice signal processing. These deviant neural mechanisms at the level of the AC and the intermediate nodes of the auditory streams might thus also affect more downstream cortical processing for decoding information in speech (left vPM) and non-speech voice signals (ACC). Our general analysis of the neural voice processing network (Fig. 2c) has shown the vPM to be specific to speech sound processing, while the ACC was specific to non-speech voice sound processing. The ACC is also a central structure in the neural empathy/mentalizing network, which points to difficulties in individuals with high autistic traits to decode the relevant social information from voice signals [55].

Taken together, our data show behavioral and especially neural processing deficits for social voice and speech signals that correlate with psychopathic and autistic traits in a community sample. Individuals scoring high on primary psychopathy showed considerable behavioral and neural processing deficits, pointing to social processing deficits at various cognitive and affective levels. Extending previous findings, our data highlight neural dysfunctions in the BG system, which might be especially relevant for decoding social information from auditory communication signals. Secondary psychopathy showed differential neural deficits, with specific and so far, unreported deficits found at the level of sensory integrative processing. This sensory deficit could be specific to voice communication signal processing, given the specific complexity of auditory signals and the neural effort to decode social information from sounds. This sensory processing deficit could be a common factor with autistic traits, for which we also found a large overlap in terms of the personality phenotype with secondary psychopathy.

## Supplementary information

Supplemental Material

## Data Availability

The data are available from the corresponding authors upon a reasonable request.
