# Supplementary material for: Psychopathic and autistic traits differentially influence the neural mechanisms of social cognition from communication signals
Source: Transl Psychiatry. 2022 Nov 29;12:494. doi: 10.1038/s41398-022-02260-x (PMC9709037; doi:10.1038/s41398-022-02260-x)
Supplement: Supplementary file 1 — Supplemental Material [file 41398_2022_2260_MOESM1_ESM.docx]

**­­­­­Psychopathic and autistic traits influence the neural mechanisms of social cognition from communication signals**

Christine Skjegstad, Caitlyn Trevor, Huw Swanborough, Claudia Roswandowitz, Andreas Mokros, Elmar Habermeyer, Sascha Frühholz

**SUPPLEMENTAL METHODS**

## Brain data acquisition

Structural and functional brain data were recorded on a 3T Philips Ingenia MR scanner by using a standard 32-channel head coil. A high-resolution structural image was acquired by using a T1-weighted scan (301 contiguous 1.2mm slices, repetition time [TR]/echo time [TE] = 1.96s/3.71ms, field of view [FOV] = 256mm, in-plane resolution 1x1mm^2^). In each participant, 407 functional whole-brain images for the sound processing experiment were recorded by using a T2*-weighted echo-planar pulse imaging (EPI) sequence (TR 1.6s, TE 30ms, flip angle [FA] 82°; in-plane resolution 220×114.2mm, voxel size 2.75×2.75×3.5mm^3^; slice gap 0.6mm) covering the whole brain. For each participant, a whole-brain magnetic field mapping sequence (TR 30ms, TEs 0.01/3.57ms, FA 60°, voxel size 2.7×2.7×4.0mm^3^) was recorded to reduce image distortions from inhomogeneities in the magnetic field. Subjects were scanned while keeping their eyes open and listening attentively to the stimuli.

## Preprocessing and statistical analysis of brain data

Preprocessing and statistical analyses of functional images were performed using the Statistical Parametric Mapping software (SPM12, Department of Cognitive Neurology, London; [www.fil.ion.ucl.ac.uk/spm).](http://www.fil.ion.ucl.ac.uk/spm).) Functional data were first manually realigned to the AC-PC axis, and functional images were then motion corrected using a 6-parameter rigid-body transformation with realignment to the mean functional image. This was followed by a slice time correction (reference slice 12) of the slices acquired within a brain volume (24 slices). Each participant’s anatomical T1 image was then co-registered to the mean functional brain image, followed by a segmentation of the T1 image for estimating normalization parameters using a geodesic shooting and Gauss-Newton optimization approach [1] for transformations into the standard space. Based on the estimated parameters, the anatomical and functional images were then normalized to the Montreal Neurological Institute (MNI) stereotactic space. Functional images were re-sampled into an isotropic 2mm^3^ voxel size during the normalization procedure. All functional images were spatially smoothed with an 8mm full-width half-maximum (FWHM) isotropic Gaussian kernel.

Functional brain data were then entered into a fixed-effects single-subject analysis, with a general linear model (GLM) design matrix containing five separate regressors for each of the five conditions plus an additional regressor for all repetition trials. All trials were modeled with a stick function aligned to the onset of each stimulus, which was then convolved with a standard hemodynamic response function (HRF). The design matrix also included six motion correction parameters as regressors of no interest to account for signal artifacts due to head motion.

Contrast images for each of the five main sound categories and from each participant were then taken to several separate random-effects factorial group-level analyses. In a first step of this analysis, we performed contrasts between conditions to determine functional brain activity patterns that were associated with general ([all voice > non-voice sounds]) and specific voice sound processing ([speech > non-voice], [non-speech > non-voice], [speech > non-speech], and [non-speech > speech]). All contrasts were thresholded at a combined voxel threshold of p<0.005 corrected for multiple comparisons at a cluster level of k=56 (maximum cluster size across all estimations performed). This combined voxel and cluster threshold corresponds to p=0.05 corrected at the cluster level and was determined by the 3dClustSim algorithm implemented in the AFNI software (<https://afni.nimh.nih.gov/afni>; version AFNI_18.3.01; including the spatial autocorrelation function [ACF] extension) according to the estimated smoothness of the data.

In a second step of the analysis, we performed a regions-of-interests (ROI) analysis of significant clusters resulting from the first factorial analysis. This analysis was done, to assess if typical brain regions that are generally associated with voice processing [2,3] show an association of their activity level with psychopathic and autistic traits. We extracted beta scores for peak location of activations (Fig. S1) and quantified the mean beta scores for all 5 experimental conditions (sound categories) in voxels of a 3mm sphere around the peak location. First, overall activation scores were calculated as the mean beta weight across all 5 conditions and correlated with personality trait scores. Second, difference beta scores between voice and non-voice conditions were then correlated to personality trait scores, using a partial correlation approach that controlled for age and gender. Age and gender were controlled to exclude potential effects of age and gender, as previous studies have found associations with voice processing regions [4,5]. To our knowledge, no other voice localizer fMRI studies have so far investigated whether general personality traits (BFI) have influence on neural voice processing. Significant correlations were determined at p=0.05 (FDR corrected). In a third step, we finally performed separate whole brain analyses to quantify the individual association between neural effects for voice processing with trait psychopathic and autistic scores across all brain voxels. Unlike the previous ROI-based analysis that was restricted to neural voice processing nodes, this third analysis analyzed the neural effects of social cognition from voices in the broader brain system outside the common neural voice processing nodes. Accordingly, separate multiple regression analyses were performed, by regressing neural activity differences for voice processing ([all voice > non-voice], [speech > non-voice], and [non-speech > non-voice])either against psychopathic trait scores (LSRPtotal, LSRPprim, LSRPsec; Fig. 3 and Fig. S4) or against autistic trait scores (AQ score; Fig. 4 and Fig. S5). These regression models also included additional regressor of no interest to control for general demographic factors (age, gender), for general personality traits (BFI), and affective traits (PANAS_ab_, STAI_trait_, BDI). For this analysis, general personality and affective traits were controlled in addition to age and gender to exclude the potential effects that such traits have on the processing of social stimuli, as found in previous studies [6–11]. Controlling these traits allows for a purer analysis on the effects from psychopathic and autistic traits on the processing of social stimuli. Results for the multiple regressions were again thresholded at a combined voxel threshold of p<0.005 and a minimum cluster level of k>55 to result in p=0.05 corrected at the cluster level.

**Additional personality trait assessments**

To assess broader personality trait patterns of the participants, we assessed additional cardinal personality traits. These additional assessments included first the Big Five Inventory (BFI, 44 items) [12] to quantify the major human personality traits of neuroticism, extraversion, openness, conscientiousness, and agreeableness. These central personality traits have been shown to influence socio-affective processing from human signals and the underlaying neural mechanisms [13,14]. Second, an affect balance score (PANAS_ab_) [15] of the participant’s general affective state and the balance between negative and positive emotions was obtained using the Positive and Negative Affect Schedule (PANAS) [16] by subtracting the negative affect (NA) score from the positive affect (PA) score after the PANAS assessment, with higher affect balance scores resulting from higher PA and lower PA [17]. Third, trait dimensions of anxiety (STAI_trait_) were assessed using the trait subscale of the State-Trait-Anxiety Inventory (STAI) [18], and fourth, trait dimensions of depressive patterns were assessed using the Beck Depression Inventory (BDI-IA) [19]. These additional personality scales were used because they might show some relationship to psychopathic and autistic traits [20–22].

**References**

1. Ashburner J, Friston KJ. Diffeomorphic registration using geodesic shooting and Gauss–Newton optimisation. NeuroImage. 2011;55:954–67.
2. Pernet CR, McAleer P, Latinus M, Gorgolewski KJ, Charest I, Bestelmeyer PEG, et al. The human voice areas: Spatial organization and inter-individual variability in temporal and extra-temporal cortices. NeuroImage. 2015;119:164–74.
3. Staib M, Frühholz S. Cortical voice processing is grounded in elementary sound analyses for vocalization relevant sound patterns. Prog Neurobiol. 2021;200:101982.
4. Ahrens M-M, Awwad Shiekh Hasan B, Giordano BL, Belin P. Gender differences in the temporal voice areas. Front Neurosci [Internet]. 2014 [cited 2021 Jun 2];8. Available from: http://journal.frontiersin.org/article/10.3389/fnins.2014.00228/abstract
5. Tremblay P, Brisson V, Deschamps I. Brain aging and speech perception: Effects of background noise and talker variability. NeuroImage. 2021;227:117675.
6. Yoon KL, Hong SW. Behavioral inhibition system sensitivity moderates audio-visual neutral information processing. J Behav Ther Exp Psychiatry. 2020;69:101597.
7. Siegel EH, Wormwood JB, Quigley KS, Barrett LF. Seeing What You Feel: Affect Drives Visual Perception of Structurally Neutral Faces. Psychol Sci. 2018;29:496–503.
8. Kreifelts B, Ethofer T, Huberle E, Grodd W, Wildgruber D. Association of trait emotional intelligence and individual fMRI-activation patterns during the perception of social signals from voice and face. Hum Brain Mapp. 2009;31:979–91.
9. Kreifelts B, Eckstein KN, Ethofer T, Wiegand A, Wächter S, Brück C, et al. Tuned to voices and faces: Cerebral responses linked to social anxiety. NeuroImage. 2019;197:450–6.
10. Karle KN, Ethofer T, Jacob H, Brück C, Erb M, Lotze M, et al. Neurobiological correlates of emotional intelligence in voice and face perception networks. Soc Cogn Affect Neurosci. 2018;13:233–44.
11. Fox E, Zougkou K. Influence of Personality Traits on Processing of Facial Expressions [Internet]. Oxford University Press; 2011 [cited 2022 Oct 26]. Available from: https://academic.oup.com/edited-volume/28040/chapter/211945247
12. John OP, Donahue EM, Kentle RL. The big five inventory: Versions 4a and 54 [Technical Report]. Berkeley Univ Calif Inst Personal Soc Res. 1991;
13. Frühholz S, Schlegel K, Grandjean D. Amygdala structure and core dimensions of the affective personality. Brain Struct Funct. 2017;222:3915–25.
14. Frühholz S, Prinz M, Herrmann M. Affect-related personality traits and contextual interference processing during perception of facial affect. Neurosci Lett. 2010;469:260–4.
15. Koydemir S, Schütz A. Emotional intelligence predicts components of subjective well-being beyond personality: A two-country study using self-and informant reports. J Posit Psychol. Taylor & Francis; 2012;7:107–18.
16. Watson D, Clark LA, Tellegen A. Development and validation of brief measures of positive and negative affect: the PANAS scales. J Pers Soc Psychol. American Psychological Association; 1988;54:1063.
17. Yamasaki K, Uchida K. Relationships between Affect and Short-Term Life Satisfaction: Considering the Activation Dimension and Balance of Affect. Int J Soc Sci Stud. 2016;4:34–40.
18. Spielberger C, Gorsuch R, Lushene R, Vagg P, Jacobs G. Manual for the state-trait anxiety inventory. Palo Alto: Consulting Psychologists Press;
19. Beck AT, Steer RA. Beck Depression Inventory: BDI. Psychological Corporation San Antonio, TX; 1993.
20. Harenski CL, Kim SH, Hamann S. Neuroticism and psychopathy predict brain activation during moral and nonmoral emotion regulation. Cogn Affect Behav Neurosci. 2009;9:1–15.
21. Miller JD, Lynam DR. Understanding Psychopathy Using the Basic Elements of Personality: Psychopathy and Personality. Soc Personal Psychol Compass. 2015;9:223–37.
22. Kim SY, Kim YA, Song D-Y, Bong G, Kim J, Kim JH, et al. State and Trait Anxiety of Adolescents with Autism Spectrum Disorders. Psychiatry Investig. 2021;18:257–65.

**SUPPLEMENTAL FIGURES**

**Fig S1. Beta estimates for regions-of-interest (ROI) for the main voice processing analysis.** Violin plots for ROIs that originated from peak locations of the main voice processing analysis as shown in Fig. 2. Abbreviations: *ACC* anterior cingulate cortex, *aST* anterior superior temporal cortex, *BLA* basolateral amygdala complex, *CMA* centromedian amygdala complex, *ioFC* infero-orbital frontal cortex, *MC* motor cortex, *mST* mid superior temporal cortex, *pMT* posterior middle temporal cortex, *PPo* planum polare, *PTe* planum temporale, *pST* posterior superior temporal cortex, *vPM* ventral premotor cortex, *nsp* non-speech sounds, *nvc* non-voice sounds, *spe* speech sounds, *voc* voice sounds.
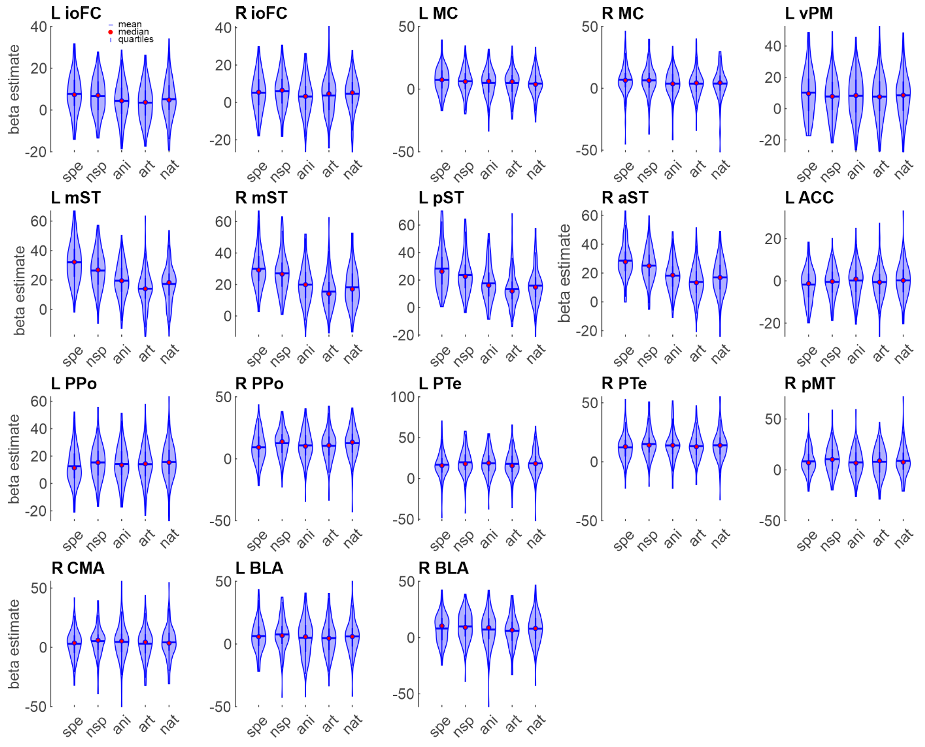


**Fig S2. Correlation analysis between personality traits and ROIs.** Pearson correlation analysis (n=113) as in Fig. 2e, but for the comparison between the voice categories, namely speech (spe) against non-speech (nsp), and vice versa. No significant correlations were found at p<0.05 (FDR corrected). Abbreviations: *AQ* Autism Spectrum Quotient, *BDI* Beck Depression Inventory, *BFI* Big Five Inventory (*extra* extraversion, *agree* agreeableness, *consc* conscientiousness, *neuro* neuroticism, *open* openness), *LSRP* Levenson Self-Report Psychopathy scale, *PANAS* Positive and Negative Affect Schedule (*ab* affective balance), *STAI* State-Trait Anxiety Inventory.


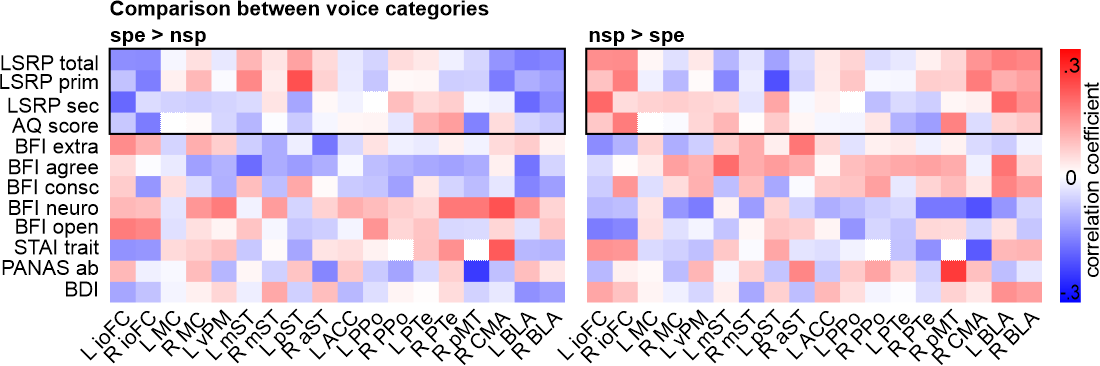


**Fig S3. Scatter plots for the correlation analysis are shown in Fig. 2e (left panel).** Correlations between personality traits and the activity difference (d beta) in the 18 ROIs based on the comparison between voice (spe, nsp) and non-voice sounds (ani, nat, art). Blue dots are single participants (n=113), black line is the linear fit based on least squared differences.


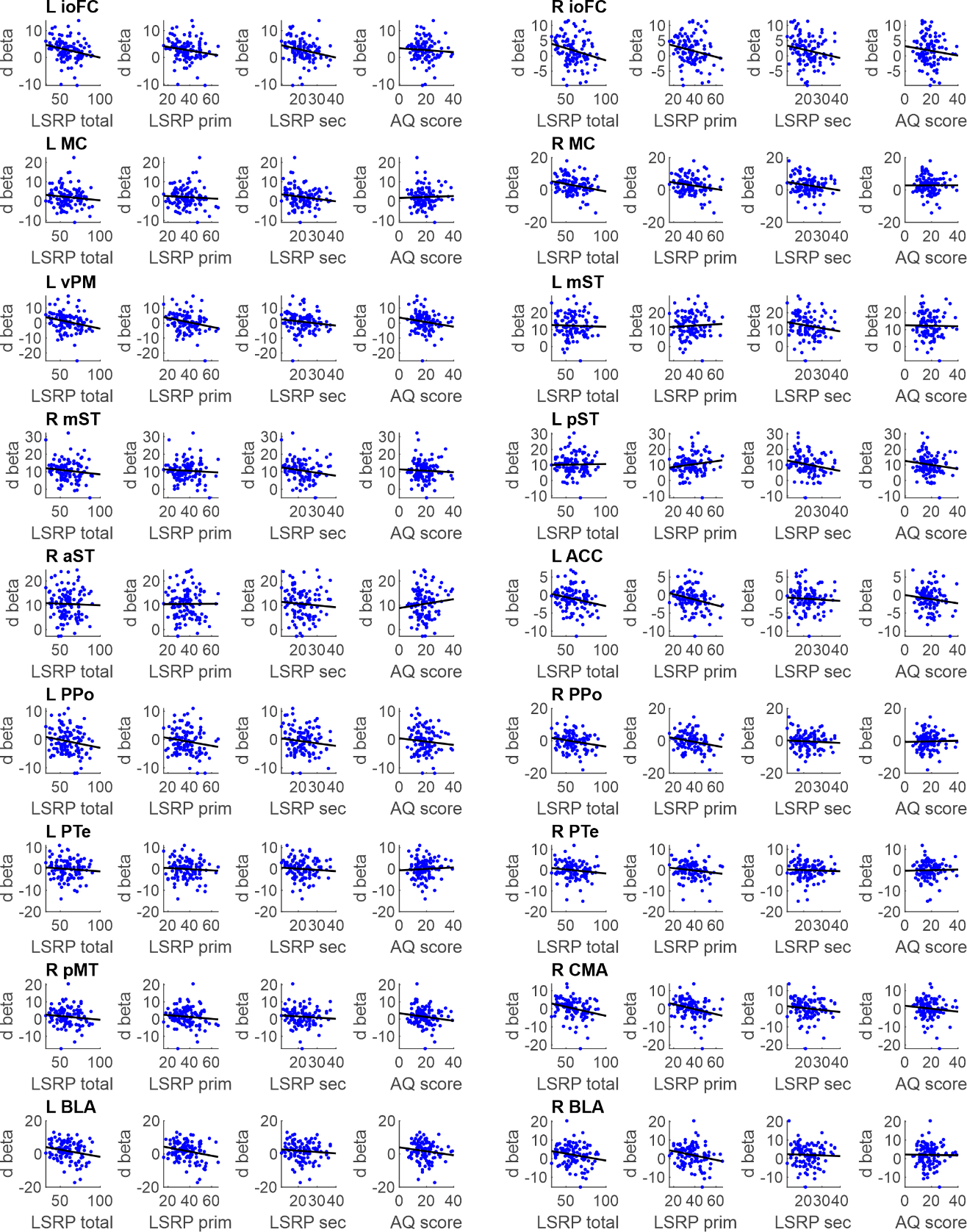


**Fig S4. Neural voice processing depending on psychopathic traits and specific voice categories.** Same analysis as in Fig. 3 (n=113), but separately for the voice categories for the (a) LSRP total score, (b) LSRP primary factor, and (c) LSRP secondary factor. All activations are thresholded at a combined voxel threshold p<0.005 and cluster level threshold k>55, resulting in at p<0.05 corrected at the cluster level. Abbreviations: *Cd* caudate nucleus, *HC* hippocampus, *Put* putamen, *nsp* non-speech sounds, *nvc* non-voice sounds, *spe* speech sounds, *voc* voice sounds.


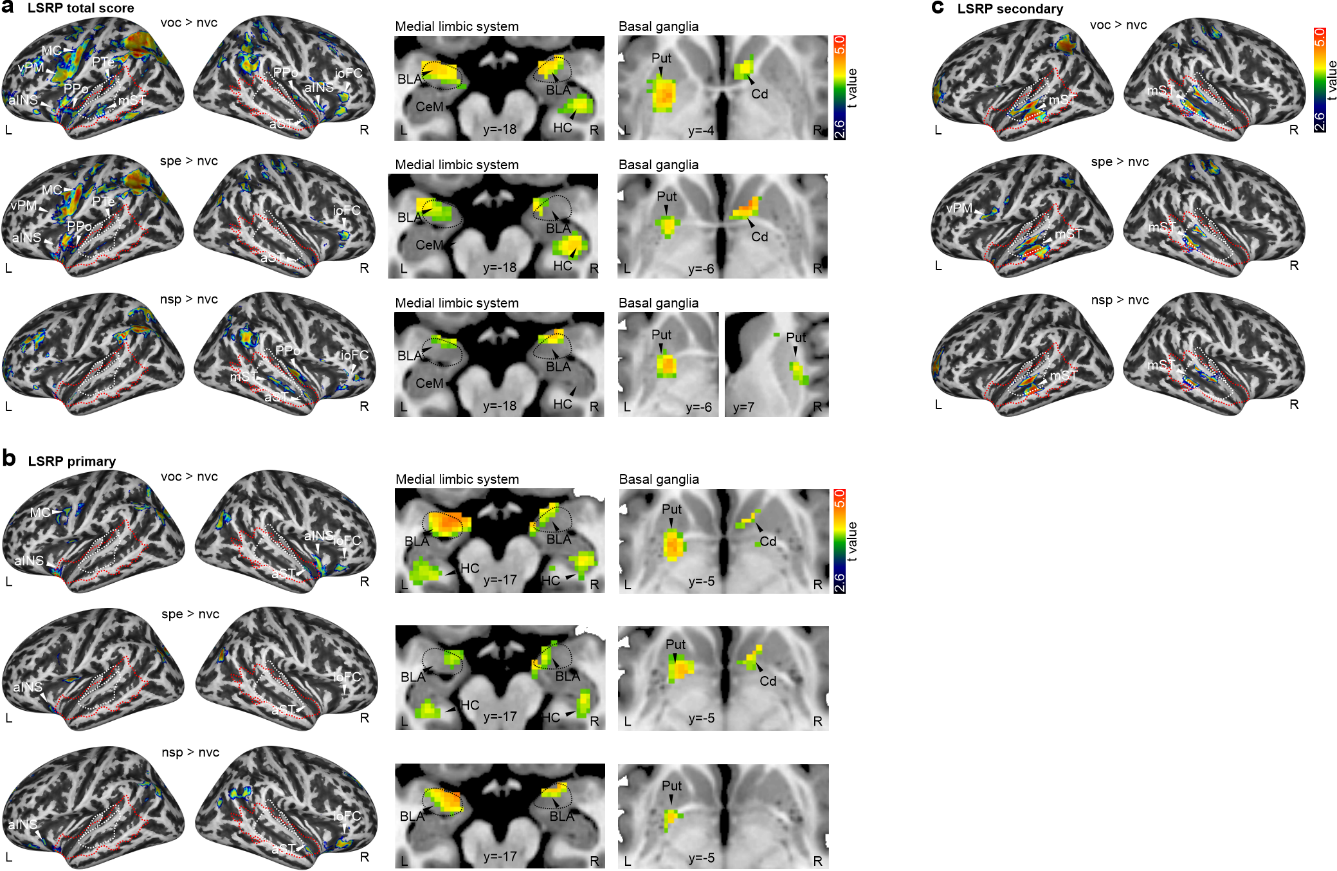


**Fig S5. Neural voice processing depending on autistic traits and specific voice categories.** Same analysis as in Fig. 4 (n=113), but separately for the voice categories. All activations are thresholded at a combined voxel threshold p<0.005 and cluster level threshold k>55, resulting in p<0.05 corrected at the cluster level.


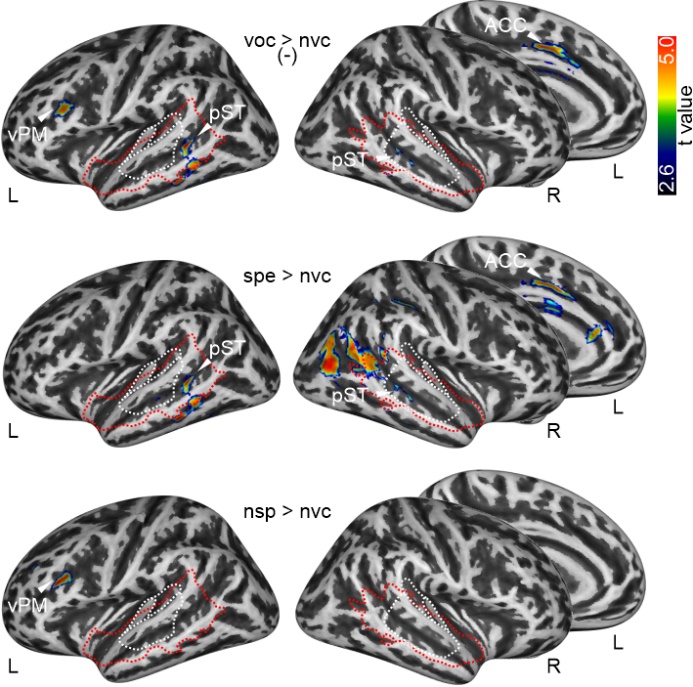


**SUPPLEMENTAL TABLES**

**Table S1. Pairwise Pearson correlation statistics for the personality traits.** Shown are the numeric Pearson correlation coefficients between the LRSP scores, the AQ score, and the additional personality traits. The table corresponds to Fig. 1e in the main text.

|  | LSRP total | LSRP prim | LSRP sec | AQ score | BFI extra | BFI agree | BFI consc | BFI neuro | BFI open | STAI trait | PANAS ab | BDI | Age | Gender |
| --- | --- | --- | --- | --- | --- | --- | --- | --- | --- | --- | --- | --- | --- | --- |
| LSRP total | 1.00 | 0.91 | 0.73 | 0.29 | -0.24 | -0.44 | -0.15 | 0.15 | -0.33 | 0.38 | -0.27 | 0.22 | -0.16 | -0.20 |
| LSRP prim | 0.91 | 1.00 | 0.37 | 0.18 | -0.14 | -0.39 | 0.04 | 0.03 | -0.34 | 0.18 | -0.19 | 0.14 | -0.16 | -0.16 |
| LSRP sec | 0.73 | 0.37 | 1.00 | 0.36 | -0.32 | -0.34 | -0.41 | 0.30 | -0.17 | 0.55 | -0.29 | 0.27 | -0.09 | -0.18 |
| AQ score | 0.29 | 0.18 | 0.36 | 1.00 | -0.44 | -0.35 | -0.15 | 0.35 | -0.23 | 0.40 | -0.05 | 0.16 | 0.03 | -0.28 |
| BFI extra | -0.24 | -0.14 | -0.32 | -0.44 | 1.00 | 0.35 | 0.23 | -0.25 | 0.30 | -0.35 | 0.22 | -0.19 | 0.04 | 0.34 |
| BFI agree | -0.44 | -0.39 | -0.34 | -0.35 | 0.35 | 1.00 | 0.21 | -0.31 | 0.36 | -0.29 | 0.26 | -0.17 | 0.04 | 0.13 |
| BFI consc | -0.15 | 0.04 | -0.41 | -0.15 | 0.23 | 0.21 | 1.00 | -0.29 | 0.22 | -0.33 | 0.34 | -0.23 | -0.05 | 0.15 |
| BFI neuro | 0.15 | 0.03 | 0.30 | 0.35 | -0.25 | -0.31 | -0.29 | 1.00 | -0.15 | 0.66 | -0.40 | 0.44 | 0.05 | 0.00 |
| BFI open | -0.33 | -0.34 | -0.17 | -0.23 | 0.30 | 0.36 | 0.22 | -0.15 | 1.00 | -0.23 | 0.20 | -0.09 | 0.06 | 0.08 |
| STAI trait | 0.38 | 0.18 | 0.55 | 0.40 | -0.35 | -0.29 | -0.33 | 0.66 | -0.23 | 1.00 | -0.50 | 0.50 | 0.08 | -0.06 |
| PANAS ab | -0.27 | -0.19 | -0.29 | -0.05 | 0.22 | 0.26 | 0.34 | -0.40 | 0.20 | -0.50 | 1.00 | -0.45 | 0.09 | 0.10 |
| BDI | 0.22 | 0.14 | 0.27 | 0.16 | -0.19 | -0.17 | -0.23 | 0.44 | -0.09 | 0.50 | -0.45 | 1.00 | 0.01 | -0.07 |
| Age | -0.16 | -0.16 | -0.09 | 0.03 | 0.04 | 0.04 | -0.05 | 0.05 | 0.06 | 0.08 | 0.09 | 0.01 | 1.00 | 0.05 |
| Gender | -0.20 | -0.16 | -0.18 | -0.28 | 0.34 | 0.13 | 0.15 | 0.00 | 0.08 | -0.06 | 0.10 | -0.07 | 0.05 | 1.00 |

**Table S2. Pairwise Pearson correlation coefficients for the personality traits and the ROIs.** Shown are the numeric values for Fig. 2d.

|  | L ioFC | R ioFC | L MC | R MC | L vPM | L mST | R mST | L pST | R aST | L ACC | L PPo | R PPo | L PTe | r PTe | R pMT | R CMA | L BLA | R BLA |
| --- | --- | --- | --- | --- | --- | --- | --- | --- | --- | --- | --- | --- | --- | --- | --- | --- | --- | --- |
| LSRP total | 0.19 | 0.06 | 0.05 | 0.02 | 0.09 | 0.04 | 0.23 | 0.06 | 0.20 | 0.03 | 0.16 | 0.08 | 0.11 | 0.27 | 0.19 | 0.13 | 0.19 | 0.15 |
| LSRP prim | 0.22 | 0.07 | 0.01 | -0.03 | 0.06 | 0.12 | 0.29 | 0.16 | 0.18 | 0.07 | 0.19 | 0.03 | 0.15 | 0.26 | 0.20 | 0.17 | 0.14 | 0.10 |
| LSRP sec | 0.05 | 0.00 | 0.09 | 0.09 | 0.09 | -0.11 | 0.02 | -0.13 | 0.12 | -0.06 | 0.04 | 0.13 | -0.01 | 0.15 | 0.10 | 0.00 | 0.18 | 0.17 |
| AQ score | 0.11 | 0.06 | 0.09 | 0.05 | 0.15 | -0.04 | -0.02 | -0.03 | 0.07 | 0.09 | 0.17 | 0.06 | 0.08 | 0.11 | 0.01 | 0.08 | 0.17 | 0.07 |
| BFI extra | 0.05 | -0.01 | -0.07 | -0.04 | -0.03 | 0.10 | 0.03 | 0.15 | 0.00 | -0.02 | -0.06 | -0.01 | 0.05 | -0.07 | -0.03 | -0.02 | -0.20 | -0.14 |
| BFI agree | -0.04 | -0.06 | -0.04 | 0.00 | -0.04 | 0.07 | -0.01 | 0.04 | -0.07 | -0.02 | -0.26 | -0.14 | -0.07 | -0.17 | -0.10 | 0.04 | -0.03 | -0.09 |
| BFI consc | -0.03 | -0.10 | 0.07 | -0.08 | -0.01 | 0.08 | 0.02 | 0.04 | -0.04 | 0.01 | -0.08 | -0.15 | -0.12 | -0.16 | -0.01 | 0.13 | -0.05 | -0.18 |
| BFI neuro | -0.06 | -0.03 | -0.02 | -0.10 | -0.11 | -0.01 | -0.04 | 0.01 | -0.02 | -0.03 | 0.11 | 0.00 | 0.05 | 0.03 | -0.10 | -0.11 | 0.12 | -0.01 |
| BFI open | -0.13 | -0.10 | -0.02 | -0.11 | -0.06 | 0.08 | -0.09 | -0.03 | -0.06 | -0.02 | -0.02 | -0.01 | 0.02 | 0.06 | 0.05 | 0.00 | -0.18 | -0.07 |
| STAI trait | -0.01 | 0.00 | -0.04 | 0.00 | 0.00 | 0.04 | 0.13 | 0.00 | 0.24 | -0.12 | 0.11 | -0.02 | 0.04 | 0.02 | 0.09 | 0.06 | 0.18 | 0.12 |
| PANAS ab | 0.14 | 0.04 | 0.10 | 0.10 | 0.16 | 0.07 | 0.05 | 0.05 | 0.00 | 0.19 | 0.00 | 0.06 | 0.15 | 0.01 | -0.03 | -0.05 | 0.02 | -0.02 |
| BDI | 0.08 | 0.01 | 0.07 | 0.03 | 0.07 | 0.08 | 0.06 | 0.15 | 0.08 | -0.08 | 0.08 | -0.01 | -0.03 | -0.03 | 0.06 | 0.07 | 0.10 | 0.05 |

**Table S3. Pairwise Pearson correlation coefficients.** Shown are the numeric values for Fig. 2e (left panel).

|  | L ioFC | R ioFC | L MC | R MC | L vPM | L mST | R mST | L pST | R aST | L ACC | L PPo | R PPo | L PTe | r PTe | R pMT | R CMA | L BLA | R BLA |
| --- | --- | --- | --- | --- | --- | --- | --- | --- | --- | --- | --- | --- | --- | --- | --- | --- | --- | --- |
| LSRP total | 0.19 | 0.06 | 0.05 | 0.02 | 0.09 | 0.04 | 0.23 | 0.06 | 0.20 | 0.03 | 0.16 | 0.08 | 0.11 | 0.27 | 0.19 | 0.13 | 0.19 | 0.15 |
| LSRP prim | 0.22 | 0.07 | 0.01 | -0.03 | 0.06 | 0.12 | 0.29 | 0.16 | 0.18 | 0.07 | 0.19 | 0.03 | 0.15 | 0.26 | 0.20 | 0.17 | 0.14 | 0.10 |
| LSRP sec | 0.05 | 0.00 | 0.09 | 0.09 | 0.09 | -0.11 | 0.02 | -0.13 | 0.12 | -0.06 | 0.04 | 0.13 | -0.01 | 0.15 | 0.10 | 0.00 | 0.18 | 0.17 |
| AQ score | 0.11 | 0.06 | 0.09 | 0.05 | 0.15 | -0.04 | -0.02 | -0.03 | 0.07 | 0.09 | 0.17 | 0.06 | 0.08 | 0.11 | 0.01 | 0.08 | 0.17 | 0.07 |
| BFI extra | 0.05 | -0.01 | -0.07 | -0.04 | -0.03 | 0.10 | 0.03 | 0.15 | 0.00 | -0.02 | -0.06 | -0.01 | 0.05 | -0.07 | -0.03 | -0.02 | -0.20 | -0.14 |
| BFI agree | -0.04 | -0.06 | -0.04 | 0.00 | -0.04 | 0.07 | -0.01 | 0.04 | -0.07 | -0.02 | -0.26 | -0.14 | -0.07 | -0.17 | -0.10 | 0.04 | -0.03 | -0.09 |
| BFI consc | -0.03 | -0.10 | 0.07 | -0.08 | -0.01 | 0.08 | 0.02 | 0.04 | -0.04 | 0.01 | -0.08 | -0.15 | -0.12 | -0.16 | -0.01 | 0.13 | -0.05 | -0.18 |
| BFI neuro | -0.06 | -0.03 | -0.02 | -0.10 | -0.11 | -0.01 | -0.04 | 0.01 | -0.02 | -0.03 | 0.11 | 0.00 | 0.05 | 0.03 | -0.10 | -0.11 | 0.12 | -0.01 |
| BFI open | -0.13 | -0.10 | -0.02 | -0.11 | -0.06 | 0.08 | -0.09 | -0.03 | -0.06 | -0.02 | -0.02 | -0.01 | 0.02 | 0.06 | 0.05 | 0.00 | -0.18 | -0.07 |
| STAI trait | -0.01 | 0.00 | -0.04 | 0.00 | 0.00 | 0.04 | 0.13 | 0.00 | 0.24 | -0.12 | 0.11 | -0.02 | 0.04 | 0.02 | 0.09 | 0.06 | 0.18 | 0.12 |
| PANAS ab | 0.14 | 0.04 | 0.10 | 0.10 | 0.16 | 0.07 | 0.05 | 0.05 | 0.00 | 0.19 | 0.00 | 0.06 | 0.15 | 0.01 | -0.03 | -0.05 | 0.02 | -0.02 |
| BDI | 0.00 | -0.02 | 0.07 | 0.01 | 0.00 | 0.05 | 0.13 | 0.05 | 0.10 | 0.07 | -0.02 | 0.02 | 0.01 | 0.07 | -0.09 | -0.10 | 0.06 | 0.01 |

**Table S4. Pairwise Pearson correlation coefficients.** Shown are the numeric values for Fig. 2e (mid panel).

|  | L ioFC | R ioFC | L MC | R MC | L vPM | L mST | R mST | L pST | R aST | L ACC | L PPo | R PPo | L PTe | r PTe | R pMT | R CMA | L BLA | R BLA |
| --- | --- | --- | --- | --- | --- | --- | --- | --- | --- | --- | --- | --- | --- | --- | --- | --- | --- | --- |
| LSRP total | -0.27 | -0.30 | -0.14 | -0.16 | -0.18 | -0.03 | -0.10 | 0.02 | -0.03 | -0.19 | -0.15 | -0.12 | -0.06 | -0.11 | -0.15 | -0.28 | -0.22 | -0.22 |
| LSRP prim | -0.19 | -0.28 | -0.08 | -0.11 | -0.17 | 0.07 | -0.05 | 0.16 | 0.00 | -0.22 | -0.15 | -0.17 | -0.06 | -0.14 | -0.14 | -0.30 | -0.21 | -0.23 |
| LSRP sec | -0.29 | -0.19 | -0.17 | -0.17 | -0.11 | -0.18 | -0.13 | -0.23 | -0.07 | -0.06 | -0.08 | 0.00 | -0.04 | -0.01 | -0.09 | -0.11 | -0.14 | -0.10 |
| AQ score | -0.12 | -0.17 | -0.01 | 0.02 | -0.10 | -0.07 | -0.01 | -0.13 | 0.07 | -0.09 | -0.02 | 0.03 | 0.07 | 0.06 | -0.21 | -0.06 | -0.10 | -0.03 |
| BFI extra | 0.07 | 0.02 | -0.06 | 0.06 | 0.01 | -0.14 | -0.08 | -0.09 | -0.20 | -0.03 | -0.05 | -0.04 | -0.03 | -0.01 | -0.01 | 0.16 | 0.08 | 0.03 |
| BFI agree | 0.18 | 0.21 | 0.02 | 0.05 | 0.04 | -0.04 | -0.05 | -0.10 | -0.08 | 0.13 | -0.09 | -0.09 | -0.04 | -0.01 | -0.04 | 0.06 | -0.06 | 0.05 |
| BFI consc | 0.17 | 0.09 | 0.14 | 0.04 | -0.07 | 0.16 | -0.01 | 0.12 | 0.10 | -0.09 | -0.01 | -0.16 | 0.13 | -0.03 | 0.00 | 0.03 | -0.12 | -0.13 |
| BFI neuro | 0.02 | -0.02 | 0.06 | 0.11 | 0.14 | 0.01 | 0.25 | -0.03 | 0.12 | 0.06 | 0.03 | 0.07 | 0.08 | 0.14 | 0.18 | 0.06 | 0.12 | 0.12 |
| BFI open | 0.17 | 0.03 | 0.01 | 0.10 | 0.02 | 0.13 | 0.02 | -0.08 | -0.08 | 0.02 | 0.03 | 0.02 | 0.04 | 0.02 | 0.09 | 0.12 | 0.05 | 0.13 |
| STAI trait | -0.16 | -0.12 | 0.07 | 0.03 | 0.09 | 0.06 | 0.11 | -0.06 | 0.16 | 0.01 | 0.01 | -0.02 | 0.11 | 0.15 | 0.04 | 0.00 | -0.01 | -0.02 |
| PANAS ab | 0.07 | 0.11 | 0.02 | 0.14 | -0.04 | 0.01 | -0.04 | 0.03 | -0.15 | 0.05 | -0.08 | -0.06 | -0.04 | -0.01 | -0.17 | 0.08 | -0.07 | -0.01 |
| BDI | -0.06 | -0.05 | 0.05 | 0.02 | 0.02 | 0.04 | 0.15 | 0.02 | 0.11 | 0.01 | -0.04 | 0.02 | 0.01 | 0.07 | -0.10 | -0.09 | -0.02 | -0.05 |

**Table S5. Pairwise Pearson correlation coefficients.** Shown are the numeric values for Fig. 2e (right panel).

|  | L ioFC | R ioFC | L MC | R MC | L vPM | L mST | R mST | L pST | R aST | L ACC | L PPo | R PPo | L PTe | r PTe | R pMT | R CMA | L BLA | R BLA |
| --- | --- | --- | --- | --- | --- | --- | --- | --- | --- | --- | --- | --- | --- | --- | --- | --- | --- | --- |
| LSRP total | -0.17 | -0.17 | -0.18 | -0.27 | -0.22 | -0.11 | -0.14 | -0.08 | -0.08 | -0.21 | -0.13 | -0.21 | -0.13 | -0.13 | -0.11 | -0.18 | -0.12 | -0.12 |
| LSRP prim | -0.15 | -0.13 | -0.14 | -0.26 | -0.24 | -0.04 | -0.08 | 0.02 | -0.05 | -0.24 | -0.11 | -0.23 | -0.09 | -0.12 | -0.09 | -0.17 | -0.18 | -0.18 |
| LSRP sec | -0.13 | -0.15 | -0.18 | -0.16 | -0.08 | -0.19 | -0.18 | -0.21 | -0.10 | -0.06 | -0.11 | -0.09 | -0.12 | -0.10 | -0.08 | -0.11 | 0.03 | 0.04 |
| AQ score | -0.07 | -0.02 | -0.01 | 0.03 | -0.08 | -0.02 | -0.01 | -0.12 | 0.10 | -0.13 | -0.05 | 0.07 | -0.04 | -0.09 | -0.06 | -0.11 | -0.08 | 0.05 |
| BFI extra | -0.10 | -0.07 | -0.03 | -0.03 | -0.07 | -0.12 | 0.01 | -0.10 | -0.09 | 0.03 | -0.12 | -0.03 | -0.01 | -0.04 | 0.00 | 0.14 | 0.03 | 0.03 |
| BFI agree | 0.18 | 0.22 | 0.05 | 0.20 | 0.17 | 0.09 | 0.05 | -0.02 | -0.01 | 0.18 | -0.01 | 0.00 | 0.11 | 0.15 | 0.07 | 0.05 | 0.12 | 0.14 |
| BFI consc | 0.14 | 0.22 | 0.15 | 0.11 | 0.02 | 0.13 | 0.07 | 0.07 | 0.12 | -0.03 | 0.08 | -0.08 | 0.14 | 0.03 | 0.08 | 0.06 | 0.01 | -0.03 |
| BFI neuro | -0.09 | -0.10 | 0.13 | 0.00 | -0.01 | 0.03 | 0.17 | 0.01 | 0.10 | -0.05 | -0.07 | 0.02 | 0.03 | -0.05 | 0.02 | -0.16 | 0.01 | 0.11 |
| BFI open | 0.01 | -0.12 | 0.06 | 0.09 | 0.01 | 0.10 | 0.04 | -0.04 | -0.04 | 0.05 | -0.14 | -0.04 | -0.06 | 0.10 | 0.14 | 0.09 | 0.11 | 0.10 |
| STAI trait | -0.04 | 0.00 | 0.05 | -0.03 | 0.03 | 0.13 | 0.12 | 0.02 | 0.17 | -0.04 | -0.01 | -0.03 | 0.05 | 0.01 | 0.04 | -0.22 | 0.09 | 0.09 |
| PANAS ab | -0.02 | 0.13 | 0.04 | 0.10 | 0.05 | 0.01 | 0.02 | -0.03 | -0.05 | -0.02 | -0.02 | 0.06 | 0.01 | -0.09 | 0.07 | 0.17 | -0.20 | -0.06 |
| BDI | 0.07 | 0.02 | 0.08 | 0.00 | -0.04 | 0.06 | 0.07 | 0.08 | 0.07 | 0.12 | 0.00 | 0.01 | 0.01 | 0.03 | -0.05 | -0.08 | 0.14 | 0.09 |

**Table S6. Pairwise Pearson correlation coefficients.** Shown are the numeric values for Fig. S2 (left panel).

|  | L ioFC | R ioFC | L MC | R MC | L vPM | L mST | R mST | L pST | R aST | L ACC | L PPo | R PPo | L PTe | r PTe | R pMT | R CMA | L BLA | R BLA |
| --- | --- | --- | --- | --- | --- | --- | --- | --- | --- | --- | --- | --- | --- | --- | --- | --- | --- | --- |
| LSRP total | -0.13 | -0.13 | -0.01 | 0.04 | -0.03 | 0.09 | 0.03 | 0.11 | 0.04 | -0.03 | -0.05 | 0.04 | 0.03 | -0.02 | -0.05 | -0.13 | -0.15 | -0.14 |
| LSRP prim | -0.07 | -0.15 | 0.02 | 0.08 | -0.01 | 0.14 | 0.02 | 0.20 | 0.05 | -0.03 | -0.07 | 0.01 | 0.01 | -0.06 | -0.06 | -0.15 | -0.10 | -0.11 |
| LSRP sec | -0.18 | -0.04 | -0.05 | -0.06 | -0.05 | -0.04 | 0.03 | -0.10 | 0.01 | -0.02 | 0.00 | 0.08 | 0.04 | 0.06 | -0.01 | -0.02 | -0.17 | -0.13 |
| AQ score | -0.06 | -0.15 | 0.00 | 0.01 | -0.05 | -0.09 | 0.00 | -0.06 | -0.01 | 0.01 | 0.01 | -0.03 | 0.09 | 0.11 | -0.15 | 0.04 | -0.05 | -0.06 |
| BFI extra | 0.13 | 0.09 | -0.05 | 0.10 | 0.06 | -0.06 | -0.10 | -0.02 | -0.16 | -0.05 | 0.03 | -0.02 | -0.02 | 0.02 | -0.02 | 0.04 | 0.06 | 0.01 |
| BFI agree | 0.04 | 0.00 | -0.02 | -0.11 | -0.09 | -0.17 | -0.10 | -0.12 | -0.10 | -0.01 | -0.08 | -0.09 | -0.10 | -0.11 | -0.10 | 0.02 | -0.16 | -0.05 |
| BFI consc | 0.06 | -0.12 | 0.04 | -0.04 | -0.09 | 0.08 | -0.08 | 0.10 | 0.00 | -0.06 | -0.07 | -0.11 | 0.03 | -0.05 | -0.08 | -0.03 | -0.14 | -0.11 |
| BFI neuro | 0.08 | 0.08 | -0.03 | 0.12 | 0.15 | -0.01 | 0.12 | -0.05 | 0.05 | 0.10 | 0.08 | 0.06 | 0.05 | 0.16 | 0.16 | 0.20 | 0.12 | 0.05 |
| BFI open | 0.15 | 0.14 | -0.04 | 0.04 | 0.01 | 0.07 | -0.01 | -0.07 | -0.06 | -0.01 | 0.12 | 0.05 | 0.07 | -0.05 | -0.04 | 0.05 | -0.03 | 0.07 |
| STAI trait | -0.13 | -0.12 | 0.04 | 0.05 | 0.07 | -0.06 | 0.00 | -0.10 | 0.03 | 0.04 | 0.01 | 0.00 | 0.07 | 0.13 | 0.00 | 0.19 | -0.08 | -0.09 |
| PANAS ab | 0.08 | -0.02 | -0.01 | 0.08 | -0.09 | 0.01 | -0.06 | 0.07 | -0.14 | 0.06 | -0.06 | -0.11 | -0.04 | 0.05 | -0.23 | -0.07 | 0.08 | 0.03 |
| BDI | -0.11 | -0.07 | -0.01 | 0.02 | 0.05 | -0.02 | 0.10 | -0.06 | 0.08 | -0.08 | -0.04 | 0.01 | 0.00 | 0.04 | -0.05 | -0.03 | -0.13 | -0.11 |

**Table S7. Pairwise Pearson correlation coefficients.** Shown are the numeric values for Fig. S2 (right panel).

|  | L ioFC | R ioFC | L MC | R MC | L vPM | L mST | R mST | L pST | R aST | L ACC | L PPo | R PPo | L PTe | r PTe | R pMT | R CMA | L BLA | R BLA |
| --- | --- | --- | --- | --- | --- | --- | --- | --- | --- | --- | --- | --- | --- | --- | --- | --- | --- | --- |
| LSRP total | 0.13 | 0.13 | 0.01 | -0.04 | 0.03 | -0.09 | -0.03 | -0.11 | -0.04 | 0.03 | 0.05 | -0.04 | -0.03 | 0.02 | 0.05 | 0.13 | 0.15 | 0.14 |
| LSRP prim | 0.07 | 0.15 | -0.02 | -0.08 | 0.01 | -0.14 | -0.02 | -0.20 | -0.05 | 0.03 | 0.07 | -0.01 | -0.01 | 0.06 | 0.06 | 0.15 | 0.10 | 0.11 |
| LSRP sec | 0.18 | 0.04 | 0.05 | 0.06 | 0.05 | 0.04 | -0.03 | 0.10 | -0.01 | 0.02 | 0.00 | -0.08 | -0.04 | -0.06 | 0.01 | 0.02 | 0.17 | 0.13 |
| AQ score | 0.06 | 0.15 | 0.00 | -0.01 | 0.05 | 0.09 | 0.00 | 0.06 | 0.01 | -0.01 | -0.01 | 0.03 | -0.09 | -0.11 | 0.15 | -0.04 | 0.05 | 0.06 |
| BFI extra | -0.13 | -0.09 | 0.05 | -0.10 | -0.06 | 0.06 | 0.10 | 0.02 | 0.16 | 0.05 | -0.03 | 0.02 | 0.02 | -0.02 | 0.02 | -0.04 | -0.06 | -0.01 |
| BFI agree | -0.04 | 0.00 | 0.02 | 0.11 | 0.09 | 0.17 | 0.10 | 0.12 | 0.10 | 0.01 | 0.08 | 0.09 | 0.10 | 0.11 | 0.10 | -0.02 | 0.16 | 0.05 |
| BFI consc | -0.06 | 0.12 | -0.04 | 0.04 | 0.09 | -0.08 | 0.08 | -0.10 | 0.00 | 0.06 | 0.07 | 0.11 | -0.03 | 0.05 | 0.08 | 0.03 | 0.14 | 0.11 |
| BFI neuro | -0.08 | -0.08 | 0.03 | -0.12 | -0.15 | 0.01 | -0.12 | 0.05 | -0.05 | -0.10 | -0.08 | -0.06 | -0.05 | -0.16 | -0.16 | -0.20 | -0.12 | -0.05 |
| BFI open | -0.15 | -0.14 | 0.04 | -0.04 | -0.01 | -0.07 | 0.01 | 0.07 | 0.06 | 0.01 | -0.12 | -0.05 | -0.07 | 0.05 | 0.04 | -0.05 | 0.03 | -0.07 |
| STAI trait | 0.13 | 0.12 | -0.04 | -0.05 | -0.07 | 0.06 | 0.00 | 0.10 | -0.03 | -0.04 | -0.01 | 0.00 | -0.07 | -0.13 | 0.00 | -0.19 | 0.08 | 0.09 |
| PANAS ab | -0.08 | 0.02 | 0.01 | -0.08 | 0.09 | -0.01 | 0.06 | -0.07 | 0.14 | -0.06 | 0.06 | 0.11 | 0.04 | -0.05 | 0.23 | 0.07 | -0.08 | -0.03 |
| BDI | 0.11 | 0.07 | 0.01 | -0.02 | -0.05 | 0.02 | -0.10 | 0.06 | -0.08 | 0.08 | 0.04 | -0.01 | 0.00 | -0.04 | 0.05 | 0.03 | 0.13 | 0.11 |

**Table S8. Functional brain activity for general voice processing.** Shown are peak locations of activity for the contrast of voice against non-voice sounds [voc > nvc] thresholded at a combined voxel threshold of p<0.005 and a cluster extend threshold of k>55, resulting in a p<0.05 corrected at the cluster level. The table corresponds to Fig. 2a in the main text.

|  |  |  | MNI |  |  |
| --- | --- | --- | --- | --- | --- |
| region | cluster size | z value | x | y | z |
| L superior temporal gyrus | 5613 | Inf | -64 | -16 | -2 |
| L inferior frontal gyrus |  | Inf | -42 | 30 | -4 |
| L inferior frontal gyrus |  | 4.01 | -46 | 14 | 20 |
| L ventral premotor cortex |  | 3.47 | -54 | 8 | 8 |
| L precentral gyrus | 73 | 6.00 | -52 | -10 | 48 |
| R superior temporal gyrus | 3881 | Inf | 64 | -6 | -6 |
| R precentral gyrus | 164 | 7.03 | 56 | -2 | 44 |
| R inferior frontal gyrus | 338 | 5.41 | 52 | 30 | 2 |
| R inferior frontal gyrus |  | 3.45 | 52 | 20 | 20 |

**Table S9. Functional brain activity for specific voice categories.** Shown are peak locations of activity for the contrasts (a) speech against non-voice sounds [spe > nvc] and (b) non-speech against non-voice sounds [nsp > nvc] thresholded at a combined voxel threshold of p<0.005 and a cluster extend threshold of k>55, resulting in at a p<0.05 corrected at the cluster level. The table corresponds to Fig. 2b in the main text.

|  |  |  | MNI |  |  |
| --- | --- | --- | --- | --- | --- |
| region | cluster size | z value | x | y | z |
| **(a) spe > nvc** |  |  |  |  |  |
| L superior temporal gyrus | 4142 | Inf | -64 | -16 | -2 |
| L superior temporal gyrus |  | Inf | -62 | -26 | -2 |
| L ventral premotor cortex |  | 4.34 | -48 | 14 | 22 |
| L precentral gyrus |  | 3.68 | -54 | 8 | 8 |
| L inferior frontal gyrus | 574 | Inf | -42 | 30 | -2 |
| R superior temporal gyrus | 3065 | Inf | 64 | -6 | -6 |
| R superior temporal gyrus |  | Inf | 62 | -16 | -4 |
| R precentral gyrus | 139 | 6.47 | 58 | -2 | 42 |
| L precentral gyrus | 85 | 5.92 | -52 | -10 | 48 |
| R inferior frontal gyrus | 72 | 3.42 | 52 | 30 | 2 |
|  |  |  |  |  |  |
| **(b) nsp > nvc** |  |  |  |  |  |
| L amygdala (BLA) |  | 3.31 | -30 | 2 | -22 |
| L superior temporal gyrus | 5254 | Inf | -64 | -18 | 0 |
| L middle temporal gyrus |  | 5.85 | -42 | 2 | -22 |
| L inferior frontal gyrus |  | 5.49 | -42 | 28 | -2 |
| L superior temporal gyrus |  | 4.28 | -56 | -48 | 22 |
| L thalamus | 58 | 3.15 | -10 | -28 | 20 |
| R superior temporal gyrus | 4426 | Inf | 64 | -8 | -6 |
| R superior temporal gyrus |  | Inf | 56 | -26 | -2 |
| R superior temporal gyrus |  | 6.64 | 54 | 12 | -18 |
| R middle temporal gyrus |  | 5.19 | 42 | 4 | -22 |
| R amygdala (BLA) |  | 3.17 | 36 | 2 | -24 |
| R amygdala (CMA) |  | 2.73 | 20 | -4 | -16 |
| R inferior frontal gyrus | 260 | 5.24 | 54 | 30 | 0 |
| R precentral gyrus | 112 | 5.22 | 56 | -2 | 44 |

**Table S10. Functional brain activity for contrasting voice categories.** Shown are peak locations of activity for the contrasts (a) speech against non-speech sounds [spe > nsp] and (b) non-speech against speech sounds [nsp > spe] thresholded at a combined voxel threshold of p<0.005 and a cluster extend threshold of k>55, resulting in at a p<0.05 corrected at the cluster level. The table corresponds to Fig. 2c in the main text.

|  |  |  | MNI |  |  |
| --- | --- | --- | --- | --- | --- |
| region | cluster size | z value | x | y | z |
| **(a) spe > nsp** |  |  |  |  |  |
| L superior temporal gyrus | 1193 | 7.62 | -62 | -14 | -4 |
| L superior temporal gyrus |  | 6.47 | -62 | -26 | -2 |
| L middle temporal gyrus |  | 3.23 | -56 | 8 | -20 |
| L ventral premotor cortex | 118 | 3.20 | -48 | 4 | 28 |
| R superior temporal gyrus | 407 | 5.59 | 64 | -4 | -8 |
| R superior temporal gyrus |  | 4.67 | 62 | -18 | -4 |
|  |  |  |  |  |  |
| **(b) nsp > spe** |  |  |  |  |  |
| L superior temporal cortex | 1406 | 4.68 | -42 | -18 | -6 |
| L planum temporale |  | 4.44 | -42 | -34 | 10 |
| L planum polare |  | 3.47 | -42 | 0 | -6 |
| L anterior cingulate cortex | 907 | 3.69 | -4 | 34 | 24 |
| R anterior cingulate cortex |  | 2.90 | 8 | 42 | 10 |
| L superior frontal gyrus |  | 3.58 | -20 | 54 | 28 |
| L medial frontal gyrus |  | 3.52 | -10 | 42 | 28 |
| R planum temporale | 2191 | 4.95 | 38 | -24 | 0 |
| R planum polare |  | 4.72 | 44 | -8 | -8 |
| R putamen |  | 3.75 | 36 | -30 | 16 |
| R superior temporal cortex |  | 3.06 | 62 | -44 | 24 |
| R amygdala (BLA) | 140 | 3.35 | 22 | 2 | -18 |
| R entorhinal cortex |  | 3.15 | 16 | -6 | -16 |
| R putamen |  | 2.78 | 22 | 0 | -6 |
| R superior temporal gyrus | 151 | 3.34 | 62 | -54 | 10 |
| R middle temporal gyrus |  | 3.24 | 56 | -54 | 4 |
| R superior frontal gyrus | 64 | 3.07 | 14 | 58 | 20 |
| R hippocampus | 172 | 3.01 | 2 | -42 | -4 |
| R hippocampus |  | 3.01 | 12 | -32 | -8 |
| R posterior cingulate cortex | 58 | 2.82 | 2 | -22 | 40 |
| R thalamus | 1174 | 4.00 | 0 | -24 | 2 |

**Table S11. Functional brain activity including LSRP_total_ as a predictor.** Shown are peak locations of activity of the multiple regression analysis with negative predictions by LSRP_total_, thresholded at a combined voxel threshold of p<0.005 and a cluster extend threshold of k>55, resulting at a p<0.05 corrected at the cluster level. The table corresponds to Fig. 3a in the main text.

|  |  |  | MNI |  |  |
| --- | --- | --- | --- | --- | --- |
| region | cluster size | z value | x | y | z |
| L putamen | 2123 | 4.26 | -22 | 2 | -8 |
| L precentral gyrus |  | 3.80 | -52 | -4 | 34 |
| L ventral premotor cortex |  | 3.40 | -54 | 2 | 18 |
| L inferior frontal gyrus |  | 3.29 | -46 | 18 | -14 |
| L anterior insula |  | 3.11 | -34 | -2 | 2 |
| L planum polare |  | 2.98 | -48 | 2 | -10 |
| L supramarginal gyrus | 3816 | 4.22 | -46 | -56 | 46 |
| L angular gyrus |  | 4.18 | -38 | -62 | 56 |
| L superior temporal gyrus |  | 3.75 | -60 | -44 | 42 |
| L supramarginal gyrus |  | 3.50 | -24 | -52 | 38 |
| L superior parietal lobule |  | 3.47 | -20 | -62 | 54 |
| L angular gyrus |  | 3.47 | -30 | -80 | 38 |
| L supramarginal gyrus |  | 3.39 | -30 | -48 | 42 |
| L angular gyrus |  | 3.32 | -28 | -64 | 22 |
| L inferior temporal gyrus | 91 | 3.33 | -56 | -64 | -12 |
| L medial frontal gyrus | 380 | 3.32 | -6 | 38 | 48 |
| L precentral gyrus | 58 | 3.30 | -34 | 8 | 40 |
| L middle temporal gyrus | 309 | 3.30 | -48 | -16 | -18 |
| L superior temporal gyrus |  | 3.14 | -58 | -20 | -12 |
| L planum temporale |  | 3.04 | -40 | -24 | -4 |
| L middle frontal gyrus | 121 | 3.29 | -38 | 42 | 30 |
| L inferior frontal gyrus | 131 | 3.26 | -22 | 64 | -4 |
| L lateral orbital gyrus |  | 3.18 | -26 | 56 | -8 |
| L inferior frontopolar gyrus |  | 2.85 | -38 | 58 | 0 |
| L amygdala (BLA) | 82 | 3.11 | -16 | -2 | 14 |
| L putamen |  | 2.67 | -18 | 10 | 10 |
| L cerebellum | 317 | 4.64 | -24 | -46 | -30 |
| R caudate nucleus | 283 | 3.98 | 8 | 2 | -16 |
| R amygdala (BLA) |  | 3.36 | 24 | 6 | -18 |
| R planum polare |  | 3.23 | 28 | 14 | -18 |
| R inferior frontal gyrus | 413 | 3.98 | 58 | 18 | 0 |
| R superior temporal cortex |  | 3.33 | 50 | 20 | -14 |
| R superior temporal cortex |  | 2.91 | 44 | 28 | -2 |
| R anterior insula |  | 2.88 | 42 | 14 | -6 |
| R superior temporal gyrus | 514 | 3.58 | 62 | -50 | 34 |
| R supramarginal gyrus |  | 3.50 | 48 | -52 | 52 |
| R superior temporal gyrus |  | 3.10 | 38 | -46 | 44 |
| R superior frontal gyrus | 94 | 3.27 | 8 | 48 | 44 |
| R medial frontal gyrus |  | 3.10 | 6 | 48 | 32 |
| R hippocampus | 129 | 3.28 | 34 | -14 | -16 |
| R planum polare |  | 3.08 | 36 | -16 | -8 |
| R middle temporal gyrus | 60 | 3.23 | 44 | 0 | -32 |
| R middle frontal gyrus | 141 | 3.08 | 32 | 14 | 46 |
| R angular gyrus | 66 | 3.08 | 32 | -82 | 42 |
| R medial frontal cortex | 72 | 3.01 | 4 | 58 | 8 |
| R angular gyrus | 67 | 2.95 | 18 | -62 | 48 |
| R superior parietal lobule |  | 2.73 | 22 | -52 | 56 |
| R supramarginal gyrus | 107 | 2.91 | 38 | -30 | 50 |
| R postcentral gyrus |  | 2.88 | 28 | -34 | 62 |

**Table S12. Functional brain activity including primary and secondary LSRP as predictors.** Shown are peak locations of activity of the multiple regression analysis with negative predictions by (a) LSRP_prim_, and (b) LSRP_sec_, thresholded at a combined voxel threshold of p<0.005 and a cluster extend threshold of k>55, resulting in at a p<0.05 corrected at the cluster level. The table corresponds to Fig. 3b in the main text.

|  |  |  | MNI |  |  |
| --- | --- | --- | --- | --- | --- |
| region | cluster size | z value | x | y | z |
| **(a) LSRP prim (-)** |  |  |  |  |  |
| L putamen | 1467 | 4.02 | -20 | 0 | -8 |
| L amygdala (BLA) |  | 4.01 | -20 | -2 | -14 |
| L anterior insula |  | 3.96 | -28 | 6 | -14 |
| L hippocampus |  | 3.93 | -4 | -10 | -16 |
| L cerebellum | 193 | 3.75 | -26 | -44 | -30 |
| L inferior temporal gyrus |  | 3.22 | -38 | -54 | -26 |
| L precentral gyrus | 96 | 3.23 | -38 | -2 | 22 |
| L angular gyrus | 213 | 3.20 | -26 | -70 | 58 |
| L supramarginal gyrus |  | 3.03 | -48 | -60 | 46 |
| L superior frontal gyrus | 61 | 3.01 | -18 | 54 | 30 |
| L precentral gyrus | 81 | 2.95 | -50 | -4 | 36 |
| L precentral gyrus |  | 2.70 | -42 | -6 | 38 |
| L hippocampus | 150 | 3.09 | -38 | -26 | -14 |
| R amygdala (BLA) | 279 | 3.40 | 24 | 6 | -18 |
| R caudate nucleus |  | 3.16 | 12 | 14 | -6 |
| R lateral orbital gyrus |  | 3.12 | 34 | 28 | -16 |
| R anterior insula |  | 3.03 | 42 | 8 | -14 |
| R superior temporal cortex |  | 2.83 | 38 | 18 | -16 |
| R superior frontal gyrus | 192 | 3.63 | 10 | 54 | 38 |
| R angular gyrus | 113 | 3.36 | 40 | -78 | 40 |
| R hippocampus | 84 | 3.21 | 38 | -16 | -18 |
| R pulvinar | 61 | 3.01 | 6 | -32 | -10 |
|  |  |  |  |  |  |
| **(b) LSRP sec (-)** |  |  |  |  |  |
| L superior temporal cortex | 470 | 3.94 | -64 | -20 | -2 |
| L middle temporal cortex |  | 3.86 | -60 | -20 | -10 |
| L superior temporal cortex |  | 3.19 | -56 | -2 | -4 |
| L superior parietal lobule | 495 | 3.54 | -30 | -50 | 42 |
| L angular gyrus |  | 3.07 | -38 | -58 | 56 |
| L supramarginal gyrus |  | 2.76 | -48 | -52 | 54 |
| L middle frontal gyrus | 207 | 3.52 | -28 | 58 | 0 |
| L inferior temporal gyrus | 84 | 3.24 | -32 | -58 | -6 |
| R superior temporal cortex | 239 | 3.78 | 64 | -28 | -2 |
| R superior temporal cortex |  | 2.99 | 68 | -36 | 4 |
| R middle temporal gyrus |  | 2.72 | 62 | -20 | -10 |
| R postcentral gyrus | 302 | 3.74 | 22 | -40 | 38 |
| R supramarginal gyrus |  | 3.37 | 32 | -46 | 34 |
| R precentral gyrus | 172 | 3.36 | 32 | -24 | 62 |
| R postcentral gyrus |  | 3.08 | 44 | -22 | 32 |

**Table S13. Functional brain activity including AQ score as a predictor.** Shown are peak locations of activity of the multiple regression analysis with negative predictions by the AQ score, thresholded at a combined voxel threshold of p<0.005 and a cluster extend threshold of k>55, resulting in at a p<0.05 corrected at the cluster level. The table corresponds to Fig. 4 in the main text.

|  |  |  | MNI |  |  |
| --- | --- | --- | --- | --- | --- |
| region | cluster size | z value | x | y | z |
| L middle temporal gyrus | 374 | 4.09 | -46 | -48 | -2 |
| L superior temporal gyrus |  | 3.16 | -60 | -48 | 8 |
| L anterior cingulate cortex | 134 | 3.22 | -14 | 2 | 36 |
| L inferior frontal gyrus | 106 | 3.11 | -36 | 20 | 20 |
| L inferior frontal sulcus |  | 2.81 | -34 | 26 | 26 |
| L ventral premotor cortex |  | 2.76 | -44 | 20 | 18 |
| R anterior cingulate cortex | 107 | 3.28 | 12 | -8 | 36 |
| R middle temporal gyrus | 62 | 3.10 | 52 | -32 | -12 |
| R superior temporal gyrus |  | 3.02 | 44 | -38 | -6 |
